# Genetic insights for enhancing conservation strategies in captive and wild Asian elephants through improved non-invasive DNA-based individual identification

**DOI:** 10.1371/journal.pone.0320480

**Published:** 2025-05-12

**Authors:** Dominic Kwesi Quainoo, Piangjai Chalermwong, Pittayarat Muangsuk, Ton Huu Duc Nguyen, Thitipong Panthum, Worapong Singchat, Trifan Budi, Prateep Duengkae, Warong Suksavate, Aingorn Chaiyes, Saowaphang Sanannu, Wanlaya Tipkantha, Nuttapon Bangkaew, Supaphen Sripiboon, Narongrit Muangmai, Kyudong Han, Patarapol Maneeorn, Mutchamon Kaewparuehaschai, Gittiyaporn Leamsaard, Chananya Kanchanasaka, Kornsorn Srikulnath

**Affiliations:** 1 Animal Genomics and Bioresource Research Unit (AGB Research Unit), Faculty of Science, Kasetsart University, Bangkok, Thailand; 2 Sciences for Industry, Faculty of Science, Kasetsart University, Bangkok, Thailand; 3 Department of Biotechnology, Faculty of Biosciences, University for Development Studies, Tamale, Ghana; 4 Interdisciplinary Graduate Program in Bioscience, Faculty of Science, Kasetsart University, Bangkok, Thailand; 5 Special Research Unit for Wildlife Genomics (SRUWG), Department of Forest Biology, Faculty of Forestry, Kasetsart University, Bangkok, Thailand,; 6 The International Undergraduate Program in Bioscience and Technology, Faculty of Science, Kasetsart University, Bangkok, Thailand; 7 Animal Conservation and Research Institute, Zoological Park Organization, Bangkok, Thailand; 8 Bureau of Conservation, Research and Education, Zoological Park Organization, Bangkok, Thailand; 9 Elephant Kingdom Project, Zoological Park Organization of Thailand, Surin, Thailand; 10 Faculty of Veterinary Medicine, Kasetsart University Kamphaeng Saen Campus, Nakhon Pathom, Thailand; 11 Department of Fishery Biology, Faculty of Fisheries, Kasetsart University, Bangkok, Thailand; 12 Bio-Medical Engineering Core Facility Research Center, Dankook University, Cheonan, Republic of Korea; 13 Department of Microbiology, Dankook University, Cheonan, Republic of Korea; 14 Department of National Parks, Wildlife and Plant Conservation, Bangkok, Thailand; 15 Biodiversity Center, Kasetsart University (BDCKU), Bangkok, Thailand; Central University of Kerala, INDIA

## Abstract

Asian elephant is a key umbrella species that plays a crucial role in maintaining biodiversity and ecological balance. As an iconic symbol of Thailand, it also significantly contributes to the nation's tourism industry. However, human activities pose serious threats to their long-term survival and population health. To tackle these challenges and develop effective conservation strategies, extensive genetic reference data were collected to enhance both captive and wild elephant conservation, improve non-invasive DNA-based individual identification, and assess genetic diversity using 18 microsatellite markers. High genetic diversity was observed across all populations; however, high levels of inbreeding were evident in NEI, EKS, BCEP, and wild elephant populations, except for the MEP population, which recorded low inbreeding levels. Significant variation in the gene pool estimates was observed across different populations, with three maternal haplogroups (α, β1, and a tentative β3) identified. A reduced panel of six microsatellite markers proved highly efficient for individual identification. Additionally, non-invasive DNA samples were tested using 18 microsatellite loci for individual identification. Using only 7 out of the 18 microsatellite loci tested, individuals were successfully identified, demonstrating enough discriminatory power to distinguish between individuals. Among these, four loci (LaT08, LaT13, FH19, and FH67) were both effective and efficient for reliable individual identification in fecal samples. These findings offer valuable insights for optimizing conservation efforts, including the design of tailored strategies to protect Asian elephants in Thailand and ensure the long-term viability of their populations.

## 1. Introduction

Asian elephant (*Elephas maximus*) holds immense ecological, cultural, and religious significance across 13 countries. The Asian elephant is categorized into three subspecies based on body size and color variations. The Sri Lankan elephant (*E. maximus maximus*) is the largest and darkest, the Indian elephant (*E. maximus indicus*) has darker skin with smaller depigmentation patches across mainland Asia, and the Sumatran elephant (*E. maximus sumatranus*) is the smallest with the lightest skin, native to Sumatra [1–4]. As an umbrella species, they contribute to vital ecological processes such as seed dispersal. However, their current range covers only 16% of their historical habitat in West, South, and Southeast Asia. The global population, estimated at 40,000–50,000 individuals, is fragmented due to poaching, habitat loss, and human-wildlife conflicts. Asian elephant is listed as endangered on the IUCN Red List and is protected under CITES Appendix I. In Thailand, human activities are the primary drivers of population decline. As of 2017, Thailand was home to an estimated 6,000–7,000 elephants, with 3,500–3,800 in captivity and 3,000–3,700 in 69 protected areas. Although the Wildlife Preservation and Protection Act of 1992 prohibits the capture of wild elephants, illegal capture persists, with calves often taken for tourism or export. This has prompted the Department of Livestock Development, Ministry of Agriculture and Cooperatives, and other agencies to implement measures to restore elephant numbers, manage captive populations, and regulate the use of wild elephants in tourist camps, supporting the country’s tourism industry. While captive elephants could potentially serve as a reservoir to augment isolated wild populations, their release also poses risks. These risks include the potential transmission of diseases to wild populations and increased human-elephant conflict due to habituated elephants exhibiting reduced fear of humans [5–7]. Subfertility is common in captive elephants, and limited breeding and inter-camp transfers may lead to increased inbreeding [8,9]. However, high genetic diversity and transfer rates between camps have also been observed [10,11,12], emphasizing the need for regular genetic assessments to guide breeding programs and legislation [10,13–15].

Meanwhile, rapid population growth and industrialization throughout Asia have intensified human-elephant conflicts, confining many elephant populations to fragmented habitats and protected areas. This isolation limits the long-term genetic diversity of the species. This reduction in adaptive capacity increases the risk of inbreeding and may impair population health. Decreased genetic diversity heightens vulnerability to diseases and reproductive issues, which threatens survival [16,17]. For instance, Salakphra Wildlife Sanctuary (14° 14′ 0.12″ N, 99° 33′ 8.95″ E), part of the Western Forest Complex, is home to around 180–200 elephants, with some estimates suggesting the population may exceed 200 [16–19]. Infrastructure development and urbanization have fragmented habitats within this complex, isolating elephant populations [20]. This has reduced genetic diversity, increased inbreeding [21], diminished the population's resilience, and exacerbated human-elephant conflicts [16], which in turn harm local agriculture. Genetic analyses using specific primers have shown variable genetic diversity within and among wild elephant populations, regardless of their size or demographic history. For instance, mitochondrial sequence analysis of elephant populations in Phuwua Wildlife Sanctuary, Taman Negara National Park, and populations in Vietnam, Laos, and the Malaysian Peninsula, which are known to be small declining populations in Southeast Asia, has revealed higher nucleotide diversity than that found in larger populations in India [14,22–24,26,29,30]. These findings are critical for conservation, as they facilitate the identification of at-risk populations and the development of targeted management strategies. Therefore, monitoring genetic diversity and determining accurate population numbers of wild elephants are essential for effective conservation management [27].

Molecular genetic methods play a key role in collecting the data needed for the management and conservation of both captive and wild Asian elephants [28]. Using microsatellite genotyping, researchers have identified significant genetic variation and potential inbreeding in both captive and wild Thai elephant populations [10,21,25,30–32]. This has led to the development of a genetic fingerprint database for Thai elephants, which supports conservation efforts by improving the management and regulation of both captive and wild populations. Genetic variability in Thai elephants has been primarily studied using blood samples; however, collecting blood from wild elephants is discouraged due to the stress it causes, the safety risks to researchers, and the high costs associated with anesthesia [33]. Consequently, non-invasive DNA sampling has become the preferred method for obtaining genomic data from elusive species [34,35]. This approach allows researchers to collect samples without disturbing the animals, thus preserving their well-being [36–38]. In elephants, fecal samples are typically used for non-invasive DNA sampling. However, the quality of DNA isolated from such samples is often insufficient for mitochondrial DNA (mtDNA) sequencing and microsatellite genotyping, which limits the accuracy of the results.

Non-invasive DNA-based individual identification is also affected by variations in DNA quality from sources such as dung, saliva, and hair, as well as the degradation of samples in various field conditions [39]. Despite these challenges, microsatellite analysis of non-invasive genetic samples has expanded the range of ecological tools available for research [40–43]. However, insufficient markers and alleles can reduce the statistical power needed to differentiate individuals, potentially leading to false recaptures in Capture-Mark-Recapture studies based on microsatellite genotyping [44]. Genotyping errors, such as allelic dropout and false alleles, can also lead to the creation of ‘ghost’ individuals, errors that result in significant overestimation of population size, sometimes inflating individual counts by as much as five times the actual population [45,46]. To address these issues, increasing the number of microsatellite markers analyzed can provide greater allele diversity, thereby improving the accuracy of individual identification through reliable genotypes. Additionally, establishing a comprehensive DNA fingerprint database for both captive and wild elephants could serve as a reference, enhancing the probability of accurately identifying individual elephants through microsatellite genotyping. In this study, we developed extensive genetic reference data, including genotypic and allelic frequencies, to enhance understanding of the genetic resources of elephants in Thailand. A research gap exists regarding the standardization of genetic markers for individual identification and genetic diversity analysis. Specifically, there is no universally accepted microsatellite panel for both blood and fecal samples from captive and wild Asian elephant populations. Therefore, the objectives are to analyze genetic diversity, evaluate microsatellite markers for identification using both sample types, standardize a co-amplifiable panel of reduced markers to minimize misidentification, and validate the panel with a larger set of published markers. With the decline in elephant numbers, evidence of inbreeding, and anthropogenic impacts across Thailand, these genetic data are expected to aid in optimizing conservation strategies for Asian elephants. This research highlights the importance of genetic information in developing tailored conservation plans, particularly considering elephants’ role in Thailand's tourism industry.

## 2. Materials and methods

### 2.1. Specimen collection and DNA extraction

**Fig 1:**
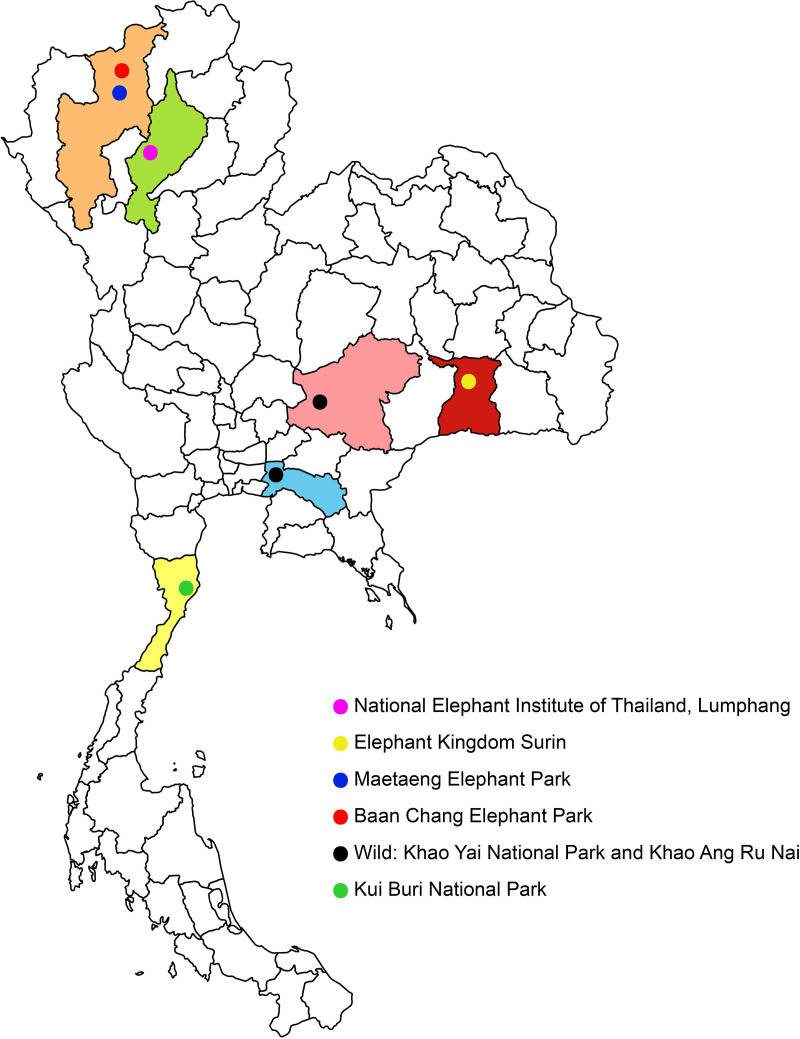
Map showing the sampling locations of captive and wild Thai Asian elephants.

Blood samples were collected from 171 individual elephants, in addition to 158 samples obtained in a previous study by Ariyaraphong et al. [30]. The collection took place between October 2020 and November 2021 from various locations, including the National Elephant Institute of Thailand (NEI, N = 93), Elephant Kingdom Surin (EKS, N = 143), Maetaeng Elephant Park (MEP, N = 46), Baan Chang Elephant Park (BCEP, N = 40), and from wild elephants in Rayong, Khao Yai, and Khao Ang Rue Nai (N = 7), (S1 Table, Fig 1). Permission for blood sample collection was granted by the relevant authorities (NEI approval no. 1400/476; ESK approval no. 1108/1036; MEP approval no. 6501.0901/3349; and 7 wild elephants sampled from Khao Ang Rue Nai, Rayong, Khao Yai National Park, Nakhon Ratchasima with Department of National Park (DNP) Permit No. 0907.404/27069). Additionally, fecal samples from Kui Buri (N = 9) were collected from Kui Buri National Park (12°08’21.2"N, 99°38’47.8"E) between March 2022 and June 2022 under DNP Permit No. 0907.404/6349. All experimental procedures were approved by the Kasetsart University Animal Experiment Committee (Approval No. ACKU63-SCI-017) and were conducted in accordance with the Regulations on Animal Experiments at Kasetsart University and the ARRIVE Guidelines (https://arriveguidelines.org).

A total of 5 mL of whole blood was collected from each elephant via the jugular vein using an 18-gauge needle attached to a 10 mL disposable syringe (NIPRO, Ayutthaya, Thailand). The blood samples were then transferred to vials containing 10 mM EDTA (Greiner Bio-One, Kremsmünster, Austria) and stored at 4°C until further processing. Genomic DNA was extracted using the standard phenol-chloroform isoamyl alcohol protocol, as described by Sambrook and Russell [47]. DNA quality and quantity were assessed using 1% agarose gel electrophoresis and a NanoDrop 2000 Spectrophotometer (Thermo Fisher Scientific, Wilmington, DE, USA). For non-invasive fecal samples, fresh fecal samples were collected from the ground shortly after defecation. Approximately 200–300g of fecal matter was placed in sterile containers and preserved in 95% ethanol for transportation and storage. The ZymoBIOMICS DNA Miniprep Kit (ZymoResearch, USA) was used to extract DNA from fecal samples. DNA quality was determined by performing 1% agarose gel electrophoresis using the MiniAmp Plus system (MiniAmp Plus, Applied Biosystems, Waltham, USA). The concentration of the extracted DNA from all fecal samples was assessed using a Nanodrop Spectrophotometer (NanoDrop 2000, Thermo Scientific, Wilmington, DE, USA).

### 2.2. Microsatellite genotyping and data analysis

Eighteen microsatellite primer sets (S2 Table) were selected from previous studies based on their polymorphic information content (*PIC*) values [48,49]. The 5’-end of the forward primer in each set was labeled with a fluorescent dye, either 6-Carboxyflourescein (6-FAM) or Hexachlorofluorescein (HEX) (Macrogen Inc., Seoul, Korea). Polymerase chain reaction (PCR) amplification was carried out following the protocol described by Ariyaraphong et al. [30]. Genetic diversity parameters, including the number of alleles per population (*N*_a_), allelic richness (*AR*), number of effective alleles (*N*_ea_), Shannon's Index (*I*), observed heterozygosity (*H*_o_), expected heterozygosity (*H*_e_), fixation index (*F*), *PIC*, *F*-statistics (*F*_IS_ and *F*_ST_), and relatedness (*r*), were calculated as outlined in Ariyaraphong et al. [30]. Additionally, *M*-ratio analysis was performed to assess historical population dynamics. Allelic dropout and false alleles were estimated using microchecker version 2.2.3 [29].

Population differentiation based on *F*_ST_, clustering analysis via principal coordinates analysis (PCoA), discriminant analysis of principal components (DAPC), and STRUCTURE analysis were conducted following Ariyaraphong et al. [30]. To better understand the genetic status of elephant populations in Thailand, microsatellite genotyping datasets from Ariyaraphong et al. [30] were retrieved and included in the analysis. The datasets were categorized into four groups: 1) NEI, 2) ESK, 3) MEP and BCEP, and 4) wild elephants from Khao Ang Rue Nai, Rayong, Khao Yai National Park and Nakhon Ratchasima. The genotypic data generated in this study were deposited in the Dryad Digital Repository (https://datadryad.org/stash/share/dNXf64BjNPjuxezpP_fF1-tLBQJd93AxJFIeGJlJuJw; accessed on 9 November 2024). A recent bottleneck event was tested using the Wilcoxon signed-rank test with a two-phase mutation model (TPM) and stepwise mutation model (SMM) to assess heterozygosity excess, given the small sample sizes of the loci. TPM and SMM were carried out with 95% single-step mutations and 5% multistep mutations, with a variance of 12 among multistep mutations.

To explore gene flow among populations, recent migration rates between captive and wild populations were estimated using a Bayesian framework in BayesASS version 3.0.5 [50]. Markov Chain Monte Carlo (MCMC) analysis was performed for 10 million generations, with a burn-in of 1 million generations, sampling every 100 generations. Posterior acceptance rates of 20%–60% were targeted to optimize parameters for migration rates (*m*), allele frequencies (*a*), and inbreeding coefficients (*F*_IS_), following Wilson and Rannala [50] guidelines. The mixing parameters were set at *a* =  0.2 and *f* =  0.2. Historical gene flow among populations was examined using MIGRATE-N version 3.6.11 [51], which estimates mutation-scaled immigration rates (*M*) and population sizes (Θ) based on coalescent theory [52]. The MCMC procedure recorded 5,000 steps every 100 generations, discarding the first 100,000 generations as burn-ins. Historical and recent genetic connectivity among populations were visualized using Circos version 0.69-8 [53]. TreeMix software version 1.12 [54] was employed to investigate demographic history, admixture, and phylogenetic relationships among populations. A consensus tree was constructed using the *consense.exe* executable from PHYLIP version 3.695 [55] and plotted using the BITE function *treemix.bootstrap* [56]. Up to ten migration events (*m*) were tested with ten iterations per event to identify the optimal number of migrations using the R package “optM” [57]. The robustness of the nodes in the TreeMix graph was assessed through 100 bootstrap repetitions at the optimal *m* value.

Microsatellite loci neutrality was tested using a Bayesian regression approach implemented in BAYESCAN [58], which estimates the probability of a locus being under selection by calculating the Bayes factor, representing the ratio of posterior probabilities of selection versus neutral models based on the data.

### 2.3. Mitochondrial D-loop sequencing and data analysis

Mitochondrial (mt) D-loop fragments were amplified using the primers MDL3 (5′-CCCACAATTAATGGGCCCGGAGCG-3′) and MDL5 (5′-TTACATGAATTGGCAGCCAACCAG-3′) [59]. PCR amplification, sequence quality control, and genetic diversity analysis based on mtDNA D-loop sequences followed the methodology detailed in Ariyaraphong et al. [30]. A statistical parsimony network of the consensus sequences and an analysis of demographic history were performed using the neutrality statistical test described in Wongtienchai et al. [60]. The sequences generated from this study have been deposited in the DNA Data Bank of Japan (DDBJ) under the accession numbers LC699890–LC700047 and LC789761–LC789924.

To investigate the haplogroups of elephant populations, multiple sequence alignments of 327 mt D-loop sequences from NEI, ESK, MEP, BCEP, and wild elephants were performed, incorporating sequence data from Ariyaraphong et al. [30] along with eight representative reference sequences for elephant haplogroups. The alignments were examined using Geneious Prime version 2023.2.1 (https://www.geneious.com). The dataset from Srikulnath et al. [61] and reference sequences for Asian elephant mtDNA haplogroups α (GenBank accession numbers: AY589513, AY589516, AY589515, and AY245817–AY245822) and β (AY589514, AY589512, AY365433, AY365432, AY245802–AY245816, AY245823–AY245827) were used to construct a haplotype network and phylogenetic tree.

Phylogenetic analysis was conducted using Bayesian inference, as implemented in MrBayes version 3.2.7a [62]. The best-fit substitution model was selected using ModelFinder [63]. The MCMC process ran simultaneously on four chains for one million generations. After stabilization of the log-likelihood value, sampling occurred every 100 generations, yielding 10,000 trees, from which a majority-rule consensus tree with mean branch lengths was generated. All initial sample points were discarded during the burn-in period. The resulting phylogenetic tree was visualized using iTOL version 6 online (https://itol.embl.de/) [64].

Bayesian coalescent-based methods were then used to assess historical demographic fluctuations through the extended Bayesian skyline plot (EBSP) method, implemented in BEAUTi version 2.0.2 (part of the BEAST version 2.0.2 package) [65,66] as described by Ariyaraphong et al. [30]. TRACER version 1.7.1 (http://beast.community/tracer) was used to evaluate the burn-in period and the effective sample sizes (ESSs) of the parameters (accessed on 24 October 2023). The EBSP method accommodates different demographic scenarios by allowing variations in population size over time. Finally, Bayesian analysis was employed to estimate migration rates and effective population sizes using coalescent theory. This was conducted with the MIGRATE-N software version 4.4.3 (Tallahassee, FL, USA) [67].

### 2.4. Construction of individual probability tests

To evaluate the effectiveness of the marker set for individual identification of Thai Asian elephants, all probabilities were calculated using GenAlEx version 6.5 [68]. The following parameters were estimated: Matching Probability (MP), Probability of Identity (P_(ID)_), Probability of Identity between Siblings (P_(ID)sibs_), and Probability of Exclusion (PE). These calculations were based on the mathematical equations provided by Patta et al. [69].

### 2.5. Marker optimization and reduced marker validation

The optimization and validation of markers followed the methodology outlined by Rasoarahona et al. [70] to select an efficient reduced microsatellite panel that enhances cost-effectiveness and reduces analysis time. From the 18 microsatellite markers initially used in the study, the panel was reduced to 6 markers based on *PIC* and the Ant Colony Optimization (ACO) algorithm developed by Iwata and Ninomiya [71], as described by Rasoarahona et al. [70]. This reduced microsatellite panel was then validated for individual identification by assessing MP, P_(ID)_, P_(ID)sibs_, and PE values. The optimized and reduced dataset was subsequently used to analyze genetic diversity, genetic differentiation, genetic clustering, and individual probability tests, as outlined in the methods described above.

### 2.6. Evaluation of the optimized marker set on a different population

The efficiency of the microsatellite panel was tested on a new set of elephant samples from Kui Buri (N = 9). Fecal samples were collected from Kui Buri National Park (12°08’21.2"N, 99°38’47.8"E) between March 2022 and June 2022 under DNP Permit No. 0907.404/6349. The optimization and validation of the reduced marker set were conducted according to the procedures outlined in Rasoarahona et al. [70]. This evaluation is expected to enhance the use of non-invasive methods, such as DNA isolated from dung samples, for individual identification and population assessment of Kui Buri elephants.

## 3. Results

### 3.1. Microsatellite data for genetic variability in the Thai Asian elephant population

Genotyping was performed on datasets from four captive elephant populations (NEI, EKS, MEP, and BCEP) as well as wild elephants. A total of 342 alleles were observed across all loci, with a mean of 14.5 ±  1.232 alleles per locus (Table 1). Significant deviations from Hardy-Weinberg equilibrium were found in all loci for the NEI and EKS populations. By contrast, certain loci in the MEP and BCEP populations did not show significant deviations. Most loci in wild elephants showed no significant departures from Hardy-Weinberg equilibrium, although evidence of linkage disequilibrium was detected. All data calculations have been deposited in the Dryad Digital Repository Dataset (https://datadryad.org/stash/share/dNXf64BjNPjuxezpP_fF1-tLBQJd93AxJFIeGJlJuJw; accessed on 9 November 2024).

**Table 1:** Genetic diversity of 329 Asian elephants (*Elephas maximus*) individuals based on 18 microsatellite loci.

Population		N	*N* ^_*a*_^	*AR*	*N* ^_*e*_^	*I*	*H* ^_*o*_^	*H* ^_e_^	*F*	*M-*ratio	*PIC*
NEI^1^	Mean	93	19.000	19.000	7.258	2.156	0.357	0.799	0.548	0.217	0.783
	SE	0.090	2.361	2.361	1.056	0.151	0.040	0.033	0.053	0.154	0.143
EKS^2^	Mean	143	27.000	27.000	9.370	2.424	0.383	0.826	0.525	0.246	0.812
	SE	1.147	3.337	3.337	1.538	0.171	0.045	0.029	0.059	0.217	0.130
MEP^3^	Mean	46	11.111	11.111	5.309	1.711	0.714	0.721	−0.018	0.629	0.184
	SE	0.000	1.777	1.777	0.928	0.159	0.047	0.041	0.067	0.318	0.876
BCEP^4^	Mean	40	11.056	11.056	5.910	1.814	0.204	0.747	0.677	0.196	0.718
	SE	0.000	1.406	1.406	1.059	0.154	0.055	0.037	0.089	0.142	0.166
Wild^5^	Mean	7	4.389	3.619	3.473	1.157	0.461	0.575	0.192	0.058	0.539
	SE	0.198	0.567	0.404	0.458	0.159	0.086	0.070	0.136	0.005	0.069
All Population	Mean	64.722	14.500	8.107	6.261	1.852	0.422	0.748	0.261	0.218	0.442
	SE	4.865	1.232	2.816	0.510	0.083	0.030	0.022	0.074	0.054	0.105

Sample size (N); number of alleles (*N*_a_); allelic richness (*AR*); number of effective alleles (*N*_e_); Shannon’s information index (*I*); observed heterozygosity (*H*_o_); expected heterozygosity (*H*_e_); *M-*ratio test (*M-*ratio); polymorphic information content (*PIC*); fixation index (*F*).

^1^NEI =  National Elephant Institute of Thailand, Lumphang. ^2^EKS =  Elephant Kingdom Surin. ^3^MEP =  Maetaeng Elephant Park. ^4^BCEP =  Baag Chang Elephant Park. ^5^Wild Elephants =  Rayong, Khao Yai and Khao Ang Rue Nai.

Null alleles were detected across all loci (LaT06, LaT08, LaT16, LaT13, LaT17, LaT24, LaT18, LaT25, LaT26, FH1, FH19, FH48, FH65, FH67, FH71, FH94, FH102, and FH103), with consistent treatment for all markers. All populations exhibited positive *F* values, except for the MEP population, which had a negative *F* value. The *PIC* values for the Thai Asian elephant populations ranged from 0.2076 to 0.9557, with Shannon's information index (*I*) ranging from 0.000 to 3.454. *H*_o_ values ranged from 0.000 to 1.000 (mean ±  SE: 0.422 ±  0.030), while *H*_e_ ranged from 0.000 to 0.957 (mean ±  SE: 0.748 ±  0.022) with wild elephant population showing zero heterozygosity at some loci (Table 1 and S3 Table). Welch’s *t*-test indicated that *H*_o_ was significantly different from *H*_e_ in the NEI, EKS, and BCEP populations but not in the MEP and wild populations. Pairwise comparisons of *H*_o_ revealed significant differences between five pairs: NEI-MEP, EKS-MEP, MEP-BCEP, MEP-wild, and BCEP-wild. Similarly, pairwise comparisons of *H*_e_ showed significant differences between MEP-wild and EKS-MEP. The *AR* across populations was 8.107 ±  2.816. Standard genetic diversity indices are summarized in Table 1 and S3 Table.

**Fig 2:**
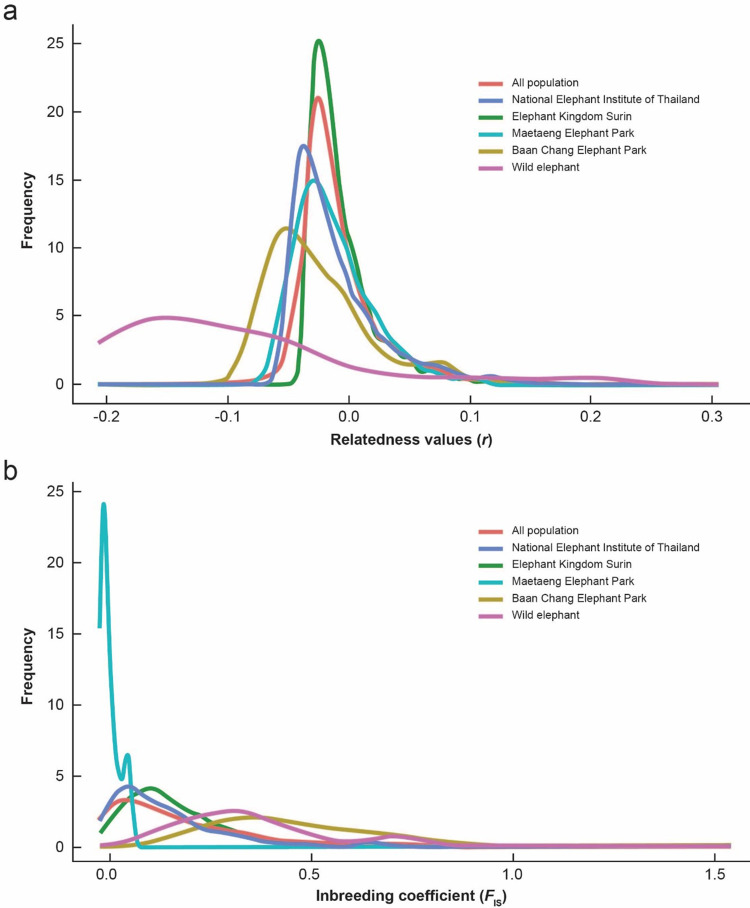
Observed distribution of (a) pairwise relatedness (r) and (b) inbreeding coefficients (FIS) for 329 Asian elephants (Elephas maximus) plotted against their respective distributions.

A pairwise relatedness test was conducted to assess genetic relationships among individuals from the five Thai Asian elephant populations. The pairwise *r* values for the 16,267 elephant combinations among 329 sampled individuals were as follows: NEI =  –0.008 ±  0.042, EKS =  –0.006 ±  0.030, MEP =  –0.012 ±  0.032, BCEP =  –0.023 ±  0.046, and wild elephants =  –0.098 ±  0.022. Of the 16,262 pairs, 99.97% had *r* values between –0.25 and 0.25, while five pairs (two from NEI and three from EKS) had *r* >  0.25. No pairs had *r* < –0.25. The distributions of *r* values were skewed left, suggesting lower-than-expected relatedness based on a null hypothesis of unrelated individuals. Pairwise *r* values across all populations showed no significant differences (Fig 2a, S4 Table). The *F*_IS_ distributions across populations were not significantly different from each other, except for the MEP population, where *F*_IS_ values were predominantly below zero (Fig 2b, S4 Table). *F*_IS_ values for individual populations ranged from –0.001 to 0.941.

The effective population size (*N*_e_) for individuals contributing genetically to the NEI population was estimated at 13.00 (95% CI: 11.9–14.2), 43.2 (95% CI: 38.9–48.1) for EKS, 157.3 (95% CI: 95.2–351.0) for MEP, 103.6 (95% CI: 73.0–169.4) for BCEP, and 11.2 (95% CI: 11.2−∞) for wild elephants. Following 110 permutations, *F*_ST_ estimates revealed significant differences (*p* <  0.05) between NEI and EKS populations; however, no significant differences were detected between captive and wild populations. Additionally, *F*_ST_^ENA^ estimates among captive populations showed no significant differences (S5 Table). AMOVA results indicated that 47% of genetic variation was among individuals within populations, while 10% was among populations (S6 Table). The largest genetic distance was observed between wild and BCEP populations, whereas NEI and EKS had the closest genetic distance based on Nei’s genetic distances and *R*_ST_ results.

**Fig 3:**
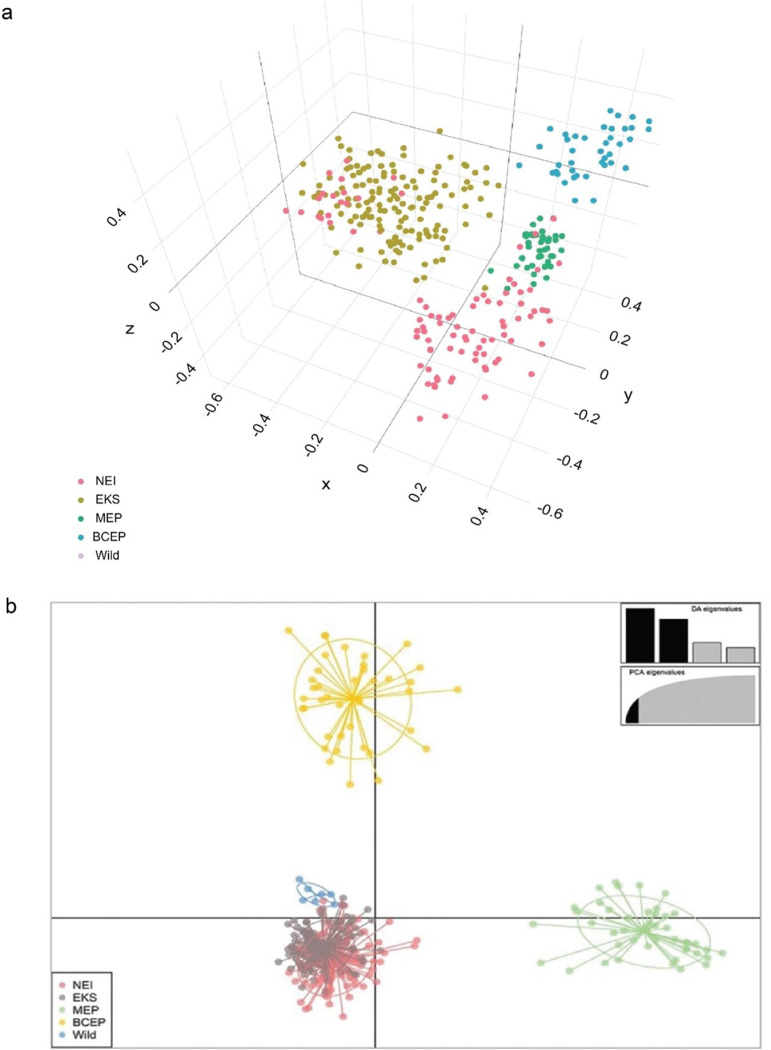
(a) Principal component analysis of Asian elephants (*Elephas maximus*) from the following locations: NEI =  National Elephant Institute of Thailand, Lampang; EKS =  Elephant Kingdom Surin; MEP =  Maetaeng Elephant Park; BCEP =  Baan Chang Elephant Park; and Wild =  wild elephants. Detailed information for all individuals is provided in S1 Table. (b) Discriminant analysis of principal components (DAPC) results. The scatter plot, based on DAPC output, shows four genetic clusters represented by different colors, with dots corresponding to individual elephants.

**Fig 4:**
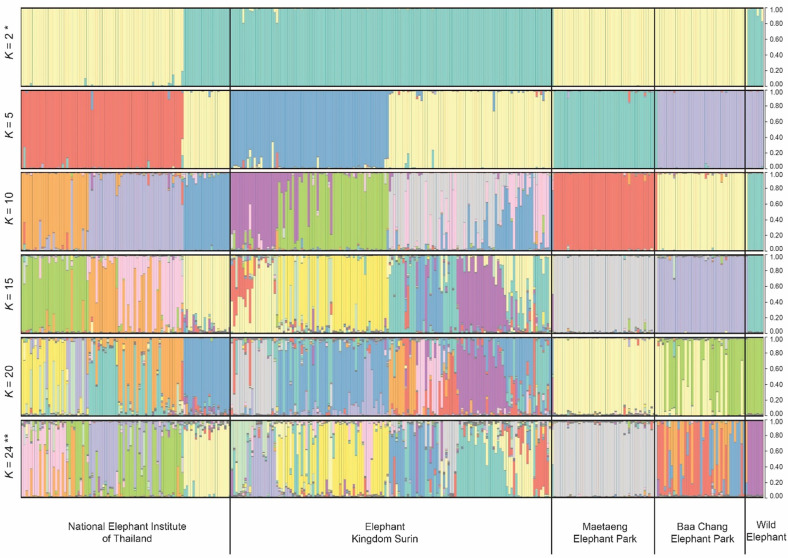
Population structure of 329 Asian elephants (*Elephas maximus*). Each vertical bar on the x-axis represents an individual elephant, while the y-axis indicates the proportion of membership (posterior probability) in each genetic cluster. Asian elephants are superimposed on the plot, with black vertical lines marking boundaries.

Principal component analysis (PCoA) of the microsatellite data showed that individual genotypes from the two clusters were separated along the first (5.94% of variation) and second (4.51% of variation) components of the PCoA (Fig 3a), which aligned with the results of DAPC. Overlap between the clusters indicated weak genetic differentiation within populations (Fig 3b). STRUCTURE analysis, using Bayesian clustering algorithms, identified various *K*-values (2, 5, 10, 15, 20, and 24) to identify the most likely and best-fitting genetic structure in the data, with *K* =  2 being the optimal clustering solution based on Evanno’s ∆ *K* and the mean ln P(*K*) showing a single peak at *K* =  24. Based on the modal value of Δ*K*, only two genetic clusters were shown in (Fig 4, S1 Fig) with NEI, MEP, and BCEP populations forming the first cluster, while the second cluster consists of EKS and wild elephants with evidence of admixture from individuals in NEI, MEP and BCEP populations whereas individuals from the NEI population contributed to all of the clusters (Fig 4, S1 Fig).

**Fig 5:**
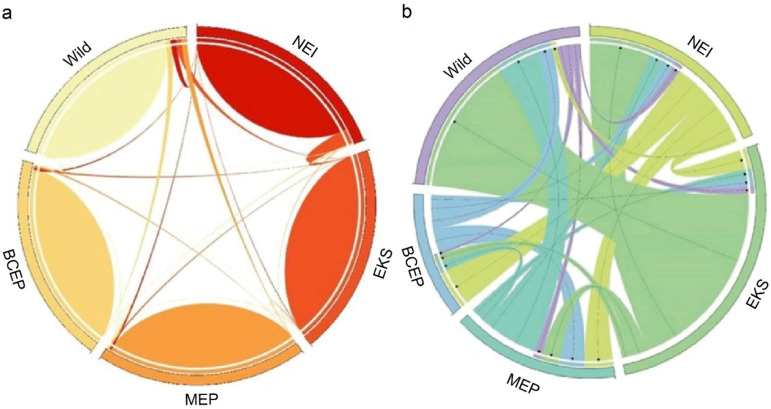
Circos visualization of source–sink migration dynamics, implemented using version 0.69-8. (a) Current migration directionality is shown using BayesAss software 3.0.5, while (b) historical migration patterns are depicted using MIGRATE-N software. The width of the migration curves indicates the relative magnitude of migration.

Recent gene flow estimates from BayesAss ranged from 0.889 to 0.991 within populations and from 0.002 to 0.075 between populations (Fig 5a, S7 Table). Wilcoxon signed-rank tests for recent bottlenecks yielded SMM values of 0.999 for NEI, EKS, and MEP, 0.995 for BCEP, and 0.032 for wild elephants. TPM values were 1.000 for NEI and EKS, 0.999 for MEP and BCEP, and 0.042 for wild elephants (indicating a normal L-shaped mode shift). *M*-ratio values for all populations were below 0.68, suggesting historical reductions in population size.

Migration rate (*M*) analysis using MIGRATE-N showed a diverse range of values based on microsatellites data from 4.333 to 299.667, with the highest rate observed from wild elephants to EKS (Fig 5b). Mutation-scaled population size (Θ) values ranged from 0.007 to 0.099 across populations, with EKS showing the highest value and MEP the lowest. Gene flow (*N*_m_) between populations ranged from 0.002 to 3.284 (S8 Table), with the highest value observed from EKS to NEI. Based on the OptM function in TreeMix, the optimal number of gene flow events was determined to be one, involving EKS and NEI (Fig S2a). After accounting for this introgression event, residuals suggested potential remaining admixture within the five populations (S2b Fig). The BAYESCAN approach identified loci LaT06, LaT08, LaT13, LaT26, and FH19 as under directional selection in captive Thai elephant populations (NEI, EKS, MEP, and BCEP), but not in wild elephants.

### 3.2. Efficiency and validation of markers for individual identification and paternity testing in different elephant populations

MP, P_(ID)_, P_(ID)sibs_, and PE were evaluated across five elephant populations (NEI, EKS, MEP, BCEP, and wild) using 18 microsatellite loci. MP was estimated for 329 Thai Asian elephants using these 18 loci. The locus-specific mean MP values ranged from 1.0 ×  10⁻¹ (FH65) to 7.4 × 10⁻² (FH103). The estimated P_(ID)_ and P_(ID)sibs_ values were similarly low, ranging from 1.0 × 10⁻¹ to 7.0 ×  10⁻³ and 2.6 ×  10⁻¹ to 4.2 ×  10⁻¹, respectively. At all 18 loci combined, the P_(ID)_ was calculated at 7.4 ×  10⁻³², and the P_(ID)sibs_ was 9.4 ×  10⁻⁹. For parentage verification where only one parent was known, the exclusion probability values ranged from 31.60% at locus FH1 to 89.79% at locus LaT08. When using all 18 microsatellite loci, the combined exclusion probability exceeded 99.8% (S3 Fig, Table 2). A comparison of the microsatellite loci, based on *PIC* and ACO algorithms (with 5% accuracy loss), was conducted to assess their efficiency in molecular testing for parentage and individual identification. This analysis allowed for a reduction in the number of microsatellite loci from 18 to 6 (LaT06, LaT08, LaT13, LaT17, LaT24, and LaT26), demonstrating the efficiency and suitability of these markers for parentage testing and individual identification (S9 Table).

**Table 2:** Matching probability (MP), Exclusion probability (PE), Probability of Identity (P_(ID)_) and Probability of Identity for sibling (P_(ID)sibs_) values per locus for 18 Microsatellites and reduced 6-marker set for 329 Asian elephant individuals.

Panel	Locus	MP	MP Combined	PE	PE Combined	P_(ID)_	P_(ID)_ Combined	P_(ID)sibs_	P_(ID)sibs_ Combined
18 loci panel	LaT06	3.53 × 10^ − 2^	1.0 × 10^ − 2^	0.872312672	0.777547529	2.2 × 10^ − 3^	3.9 × 10^ − 2^	2.7 × 10^ − 1^	3.4 × 10^ − 1^
	LaT08	1.06 × 10^ − 2^	1.0 × 10^ − 2^	0.897936279	0.965098334	1.4 × 10^ − 3^	3.9 × 10^ − 2^	2.6 × 10^ − 1^	3.4 × 10^ − 1^
	LaT16	1.08 × 10^ − 2^	6.2 × 10^ − 4^	0.780548572	0.987927136	7.0 × 10^ − 3^	1.9 × 10^ − 3^	2.8 × 10^ − 1^	1.2 × 10^ − 1^
	LaT13	1.16 × 10^ − 2^	3.9 × 10^ − 4^	0.812001112	0.997293577	5.1 × 10^ − 3^	7.9 × 10^ − 4^	2.8 × 10^ − 1^	7.8 × 10^ − 2^
	LaT17	4.33 × 10^ − 2^	2.4 × 10^ − 5^	0.579874479	0.998774643	3.2 × 10^ − 2^	3.8 × 10^ − 5^	3.3 × 10^ − 1^	2.7 × 10^ − 2^
	LaT24	2.61 × 10^ − 2^	4.3 × 10^ − 6^	0.754117323	0.999612844	9.1 × 10^ − 3^	5.9 × 10^ − 6^	2.9 × 10^ − 1^	1.2 × 10^ − 2^
	LaT18	2.37 × 10^ − 2^	4.3 × 10^ − 6^	0.803073461	0.999885577	5.5 × 10^ − 3^	4.0 × 10^ − 7^	2.8 × 10^ − 1^	4.4 × 10^ − 3^
	LaT25	4.86 × 10^ − 2^	2.7 × 10^ − 7^	0.842348541	0.999979865	3.5 × 10^ − 3^	1.9 × 10^ − 8^	2.7 × 10^ − 1^	1.5 × 10^ − 3^
	LaT26	4.40 × 10^ − 2^	3.7 × 10^ − 9^	0.887041159	0.999995775	1.7 × 10^ − 3^	1.2 × 10^ − 9^	2.7 × 10^ − 1^	5.5 × 10^ − 4^
	FH01	1.11 × 10^ − 1^	9.4 × 10^ − 10^	0.315961534	0.999996998	1.2 × 10^ − 1^	1.6 × 10^ − 10^	4.3 × 10^ − 1^	2.3 × 10^ − 4^
	FH19	1.27 × 10^ − 1^	9.4 × 10^ − 10^	0.337162518	0.999997582	1.0 × 10^ − 1^	1.6 × 10^ − 10^	4.2 × 10^ − 1^	2.3 × 10^ − 4^
	FH48	3.58 × 10^ − 2^	3.1 × 10^ − 10^	0.582063841	0.999998870	3.1 × 10^ − 2^	3.9 × 10^ − 11^	3.3 × 10^ − 1^	1.2 × 10^ − 4^
	FH65	1.02 × 10^ − 1^	7.7 × 10^ − 11^	0.356055583	0.999999252	1.0 × 10^ − 1^	8.3 × 10^ − 12^	4.1 × 10^ − 1^	6.0 × 10^ − 5^
	FH67	6.69 × 10^ − 2^	2.2 × 10^ − 11^	0.460447768	0.999999545	6.0 × 10^ − 2^	1.8 × 10^ − 12^	3.6 × 10^ − 1^	3.0 × 10^ − 5^
	FH71	6.36 × 10^ − 2^	6.3 × 10^ − 12^	0.531147900	0.999999632	4.1 × 10^ − 2^	3.3 × 10^ − 13^	3.4 × 10^ − 1^	1.4 × 10^ − 5^
	FH94	2.39 × 10^ − 2^	3.2 × 10^ − 12^	0.678985164	0.999999865	1.7 × 10^ − 2^	1.4 × 10^ − 13^	3.0 × 10^ − 1^	9.2 × 10^ − 6^
	FH102	8.17 × 10^ − 2^	1.6 × 10^ − 13^	0.525543158	0.999999923	4.3 × 10^ − 2^	1.4 × 10^ − 14^	3.4 × 10^ − 1^	3.6 × 10^ − 6^
	FH103	7.40 × 10^ − 2^	1.6 × 10^ − 13^	0.427489060	0.999999955	7.0 × 10^ − 2^	1.4 × 10^ − 14^	3.7 × 10^ − 1^	3.6 × 10^ − 6^
reduced 6 loci panel	LaT06	3.54 × 10^ − 2^	1.1 × 10^ − 3^	0.8715838	0.8715838	2.24 × 10^ − 3^	2.3 × 10^ − 3^	2.68 × 10^ − 1^	2.7 × 10^ − 1^
	LaT08	1.06 × 10^ − 2^	6.2 × 10^ − 6^	0.8979363	0.9868934	1.39 × 10^ − 3^	3.1 × 10^ − 6^	2.64 × 10^ − 1^	7.1 × 10^ − 2^
	LaT13	1.16 × 10^ − 2^	3.0 × 10^ − 8^	0.8120011	0.9975360	5.06 × 10^ − 3^	1.6 × 10^ − 8^	2.77 × 10^ − 1^	2.0 × 10^ − 2^
	LaT17	4.33 × 10^ − 2^	1.8 × 10^ − 9^	0.5798745	0.9989648	3.22 × 10^ − 2^	5.1 × 10^ − 10^	3.28 × 10^ − 1^	6.4 × 10^ − 3^
	LaT24	2.61 × 10^ − 2^	3.5 × 10^ − 11^	0.7541173	0.9997455	9.09 × 10^ − 3^	4.6 × 10^ − 12^	2.88 × 10^ − 1^	1.9 × 10^ − 3^
	LaT26	4.40 × 10^ − 2^	3.5 × 10^ − 11^	0.8870412	0.9999712	1.72 × 10^ − 3^	7.9 × 10^ − 15^	2.65 × 10^ − 1^	4.9 × 10^ − 4^

The six optimized loci exhibited MP values ranging from 1.1 ×  10⁻² (LaT08) to 4.4 ×  10⁻² (LaT26). The P_(ID)_ and P_(ID)sibs_ values for these six loci ranged from 1.4 ×  10⁻³ to 9.1 ×  10⁻³ and 2.6 × 10⁻¹ to 3.3 × 10⁻¹, respectively. When combined, the six loci produced a P_(ID)_ of 7.9 × 10⁻^15^ and a P_(ID)sibs_ of 7.1 × 10⁻². For parentage verification, with only one parent known, exclusion probability ranged from 57.99% at locus LaT17 to 89.79% at locus LaT08. The combined probability of exclusion for the six loci reached 99.99% efficacy (S4 Fig, Table 2).

### 3.3. Marker validation for individual identification and parentage testing on non-invasive samples

To validate matching profiles, fecal samples from Kui Buri elephants were added to the dataset, which already included blood samples from NEI, EKS, MEP, BCEP, and wild elephants, for individual identification and parentage testing. These samples were analyzed using 18 microsatellite markers. The locus-specific mean MP values ranged from 6.0 ×  10⁻² (FH67) to 1.0 ×  10⁰ (FH1). The estimated P_(ID)_ and P_(ID)sibs_ for each locus ranged from 4.8 ×  10⁻² to 1.0 ×  10⁰ and 3.5 ×  10⁻¹ to 1.0 ×  10⁰, respectively.

For the combined 18 loci, the P_(ID)_ value was 3.8 ×  10⁻¹ and the P_(ID)sibs_ value was 5.9 ×  10⁻¹. In cases of parentage verification where only one parent was known, the lowest exclusion probability was observed at locus FH1 (0.00%), while the highest was recorded at locus LaT25 (50.12%). The exclusion probability for the combined 18 microsatellite loci was 0.995, indicating a 99.5% likelihood of correctly excluding a non-parent (S5 Fig, S10 Table).

### 3.4. Genetic variability of Thai Asian elephant population based on mitochondrial haplotype analysis

**Fig 6:**
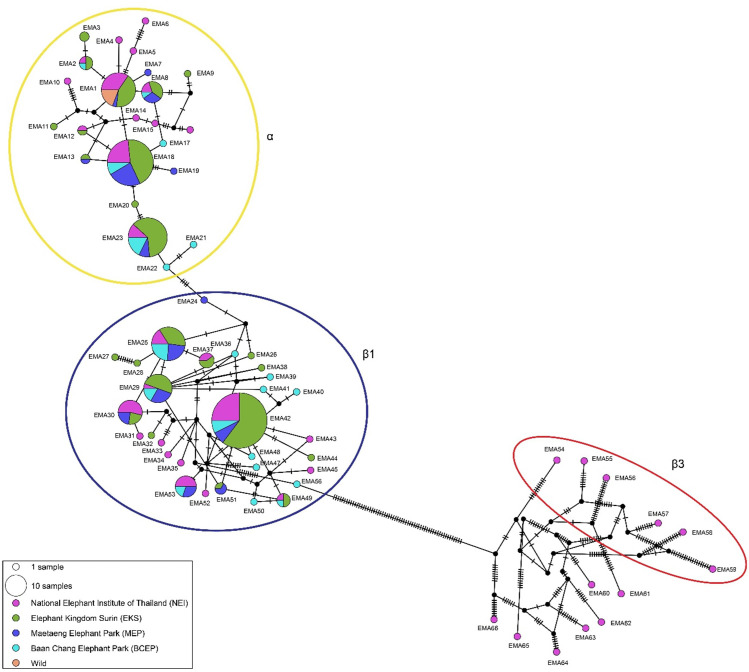
Haplotype network based on mitochondrial D-loop sequence data from Asian elephants (*Elephas maximus*) at the National Elephant Institute of Thailand (NEI, Lampang), Elephant Kingdom Surin (EKS, Surin), Maetaeng Elephant Park (MEP, Chiang Mai), Baan Chang Elephant Park (BCEP, Chiang Mai), and wild elephants. EMA =  haplotype.

The amplified length of the mtDNA D-loop sequences was 640 bp, with an alignment length of 380 bp, revealing 66 distinct haplotypes. The haplotype and nucleotide diversities were calculated as 0.906 ±  0.008 and 0.040 ±  0.005, respectively (Table 3). A complex haplotype network was constructed due to the large number of polymorphic sites and haplotypes detected. Multiple sequence alignments of 321 elephant mtDNA D-loop sequences were grouped into haplogroups α and β, with β further subdivided into β1 and a tentative β3 (Fig 6). Of the total elephant population, 29.79% were classified under the α haplogroup, 50.15% under the β1 sub-clade, and 3.95% under the tentative β3 sub-clade. Additionally, two elephants, CBC101 and MEP256, shared sequences with elephants from India and Sri Lanka, which belong to a different subspecies (*E. maximus maximus*) of the Asian elephant.

**Table 3:** Mitochondrial D-loop sequence diversity for Asian elephants (*Elephas maximus*).

Population	N	Number of haplotypes (H)	Theta (per site) from *S*	Average Number of Nucleotide Differences (*k*)	Overall Haplotype	Nucleotide diversities (π)
NEI^1^	92	40	0.117	30.202	0.935 ± 0.014	0.085 ± 0.014
ESK^2^	140	24	0.017	6.804	0.858 ± 0.018	0.019 ± 0.001
MEP^3^	46	14	0.017	7.888	0.900 ± 0.024	0.022 ± 0.001
BCEP^4^	40	20	0.024	7.779	0.936 ± 0.019	0.022 ± 0.002
Wild^5^	5	1	0.000	0.000	0.000 ± 0.000	0.000 ± 0.000
All populations	323	66	0.103	14.337	0.906 ± 0.008	0.040 ± 0.005

^1^NEI =  National Elephant Institute of Thailand, Lumphang.

^2^EKS =  Elephant Kingdom Surin.

^3^MEP =  Maetaeng Elephant Park.

^4^BCEP =  Baag Chang Elephant Park.

^5^Wild Elephants =  Rayong, Khao Yai and Khao Ang Rue Nai.

The *F*_ST_ values for the mtDNA D-loop sequences ranged from 0.000 to 0.483, *G*_ST_ values ranged from 0.001 to 0.138, and *Φ*_ST_ values ranged from 0.005 to 0.174. *N*_m_ values ranged from 0.535 to infinity, *D*_xy_ values ranged from 0.020 to 0.059, and *D*_a_ values ranged from 0.000 to 0.019. All calculations have been deposited in the Dryad Digital Repository Dataset: https://datadryad.org/stash/share/dNXf64BjNPjuxezpP_fF1-tLBQJd93AxJFIeGJlJuJw (accessed on 9 November 2024). A phylogenetic tree demonstrated that individuals from the five populations were closely related and grouped into distinct clusters, which were separate from those of other species (S6 Fig). The Extended Bayesian Skyline Plots (EBSPs) based on the mtDNA D-loop sequences showed consistent population changes across all studied populations which suggest that these populations have experienced similar evolutionary pressures over a period of time (S7 Fig).

## 4. Discussion

### 4.1. Gene pools and the effects of different selective pressures in captive and wild populations

Genetic diversity is crucial for the adaptive evolution of populations, allowing them to respond to environmental changes. This study revealed significantly higher genetic diversity, measured through microsatellites, compared to previous studies on captive and wild Asian elephants. This increase is likely due to the larger sample size used [29,30,72,75]. Interestingly, captive populations exhibited greater genetic diversity than wild populations, based on both microsatellite genotyping and mtDNA sequences. The mtDNA haplotypes were grouped into α and β haplogroups, with the β haplogroup further subdivided into β1 and a tentative β3 sub-clade, consistent with findings by Srikulnath et al. [61]. The β3 subclade consists of rare, non-shared haplotypes with significant pairwise differences, which may seem inconsistent within the haplotype network. However, this pattern is consistent with the evolutionary history of Asian elephant mitochondrial lineages, as population isolation led to the formation of β1, β2, and putative β3 subclades [30,75]. Two sequences in this study showed genetic similarities with elephants from India and Sri Lanka, suggesting historical migration or gene flow between these populations [75].

The presence of numerous haplotypes in captive populations indicates that collaboration and genetic exchange among camps have been effective. These exchanges, along with diverse origins, are likely to enhance the adaptability of captive elephants through improved management practices, contributing to the genetic database for future conservation programs [76]. By contrast, wild populations face challenges from habitat fragmentation, which exacerbates genetic drift and reduces genetic variability [30,72–74,77,78]. While high genetic diversity was observed overall, the higher *H*_e_ compared to *H*_o_ suggests potential inbreeding in Thai elephant populations, as confirmed by *F*_IS_ and *r* values [30]. This inbreeding may be driven by ongoing habitat fragmentation, which predominantly affect the wild elephant populations [78,79]. Thailand has been identified as a hotspot for elephant diversity [74], yet alarming rates of habitat loss and fragmentation, coupled with strong inbreeding signals, put these populations at increased risk of local extinction, threatening their long-term viability and adaptability. Among the populations studied, the MEP exhibited the lowest inbreeding levels and the highest *N*_e_, reflecting successful breeding management [30]. By contrast, other populations showed higher inbreeding coefficients and lower *N*_e_ values, suggesting that genetic drift and habitat isolation have historically influenced these populations. This aligns with *M*-ratio test results, which indicate past reductions in population size due to long-term bottleneck events. However, recent bottlenecks were not detected in captive populations, consistent with previous studies [30,80]. Neutrality tests, such as Tajima’s *D*, further support this, suggesting that management practices, including frequent transfers of individuals between camps, have maintained genetic stability [12,29,72,81]. The low genetic differentiation (*F*_ST_ values) and results from AMOVA confirm that most genetic variation occurs within populations.

By contrast, evidence of recent bottlenecks was observed in the wild population, as indicated by Wilcoxon signed-rank tests (*p* <  0.05). However, Tajima’s *D* value of 0 suggests a stable population structure, supported by ESBP results, which indicate that the evolutionary processes influencing population size changes are similar across both captive and wild populations. This apparent stability may be influenced by ongoing habitat fragmentation in Thailand’s wild environments. The reduced genetic diversity and effective population size observed in wild populations are likely the result of bottleneck events, as previously documented [12,72,81,82]. However, the small sample size for the wild population (N = 7) may have biased Tajima's *D* results, influenced by sampling methodology, timing of the bottleneck, genetic drift, mutation rates, or model assumptions [83]. Additionally, the wild elephants were sampled from different locations, which may further complicate the interpretation of bottleneck effects.

Distinct genetic clustering was identified for the BCEP and MEP populations through DAPC and PCoA analyses. Bayesian clustering also supported this, indicating divergent origins or restricted gene flow between BCEP, MEP, and other populations. NEI and EKS populations, on the other hand, showed high gene flow and introgression, likely due to policies at these government institutions that allow elephants to be recruited from individual owners or donors. Frequent transfers of individuals or semen, particularly for breeding males, may explain this high connectivity. A partial genetic relationship between the wild and EKS populations was also identified, possibly reflecting historical gene flow linked to past capture and trade practices that supplied elephants for Thailand’s tourism industry.

Microsatellite genotyping offers an effective tool for identifying individuals and preventing illegal trade [30,76,84,85]. Using the BAYESCAN approach, loci LaT06, LaT08, LaT13, LaT26, and FH19 were identified as being under directional selection in captive populations (NEI, EKS, MEP, and BCEP) but not in wild populations. This indicates that captive breeding programs may exert different selective pressures compared to natural environments, potentially leading to the development of traits that differ from those seen in wild populations [86]. Such selective pressures may result in linkage disequilibrium, where certain alleles are favored in captive environments but may be maladaptive in the wild. This is further supported by significant deviations from Hardy-Weinberg equilibrium in captive populations, while the wild population appears to remain in equilibrium. Specific alleles, such as FH103-158, LaT24-189, and LaT08-219, were observed only in the wild population, whereas allele LaT18-274 was specific to captive populations. Additionally, allele LaT13-219 had a high frequency (0.79) in wild populations but was low in captive populations. This suggests that different selective pressures may be acting on wild and captive populations. However, the lack of comprehensive data limits the interpretation of directional selection in captive elephant populations in Thailand.

### 4.2. Individual identification and parentage testing for both invasive and non-invasive sampling

This study evaluated captive and wild elephant populations in Thailand using microsatellite genotyping for individual identification and parentage verification. The microsatellite markers used were highly polymorphic, with *PIC* values for each population exceeding 0.5 [87]. This high variability highlights the effectiveness of these markers for accurate individual identification and parentage testing as these markers are cost-effective and suitable for implementing action plans in a local context as compared to Whole Genome Sequencing (WGS)/Single Nucleotide Polymorphism (SNP) panels [88,89]. The probability of identity P_(ID)_ was used to assess the likelihood that two randomly chosen individuals would share identical genotypes across multiple loci. In natural populations, P_(ID)sibs_ provide an upper limit for co-dominant loci [90]. Parentage exclusion probability (PE) was calculated using genotypic data to measure the likelihood of excluding an unrelated candidate parent, ensuring reliable parentage testing. Previous studies have recommended threshold values for P_(ID)_ ranging from 10⁻³ to 10⁻³ and for P_(ID)sibs_ <  2 ×  10⁻² for wildlife forensic applications [91,92].

In this study, the average P_(ID)_ value across 18 microsatellite markers was 7.4 ×  10⁻³², indicating a very low probability of two individuals sharing identical genotypes by chance, which emphasizes the robustness of these markers in identifying and tracking elephants. This is considered highly sufficient for individual identification in Asian elephants in Thailand [93]. Similarly, the P_(ID)sibs_ value was 9.4 ×  10⁻^9^, demonstrating the markers’ ability to distinguish between individuals or siblings [94]. The markers also displayed a high PE rate of 99.8%, confirming their capacity to exclude unrelated candidate parents accurately [95]. Thus, the use of 18 microsatellite loci in this study was sufficient for distinguishing individuals, even among closely related elephants. To enhance cost and time efficiency while maintaining adequate discrimination power, ACO algorithms were applied to reduce the microsatellite marker panel from 18 loci. A reduced panel of six markers performed efficiently across all identification parameters, showing a *PIC* value of 0.71 and a PE rate of 99.99%, with an error threshold of less than 5%. The 6-marker panel also produced a P_(ID)_ value of 7.9 × 10⁻^15^, further supporting its use in individual identification, particularly in conservation efforts aimed at preventing illegal trade.

In the wild population, genetic monitoring relied on non-invasive sampling [96]. Individual identification through microsatellite genotyping was assessed using P_(ID)_ and P_(ID)sibs_ values. Thresholds of P_(ID)sibs_ <  0.05 and P_(ID)_ <  0.001–0.0001 are commonly applied to distinguish individuals within a population [90,96]. In this study, 18 microsatellite loci from wild elephants demonstrated sufficient power to differentiate individuals, yielding P_(ID)_ =  6.7 ×  10⁻^14^ and P_(ID)sibs_ =  3.2 ×  10⁻^6^. However, the low DNA quality from non-invasive samples hindered successful genotyping. Only seven loci (LaT08, LaT13, FH19, FH67, FH71, FH102, and FH103) were successfully genotyped in nine wild elephants. Despite this, the P_(ID)_ and P_(ID)sibs_ values from these seven loci (P_(ID)_ =  1.1 ×  10⁻^6^, P_(ID)sibs_ =  3.9 ×  10⁻³) met the required thresholds [90,96] with no matching multi-locus genotypes detected. The analysis suggested that a minimum of three loci is needed to reach the P_(ID)_ threshold of <  0.001, while four loci are required to meet the P_(ID)sibs_ threshold of <  0.05 [96]. The loci LaT08, LaT13, FH19, and FH67 provided the lowest individual P_(ID)_ and P_(ID)sibs_ values and should be prioritized in future studies. A combination of these four loci achieved an accuracy rate of ≥ 99% (PE) for individual identification in wild populations. This suggests that the nine fecal samples analyzed in this study came from distinct, unrelated individuals. However, identification based on microsatellite loci can have an error rate of 1% to 2.5% [97,98] in elephants due to factors such as allelic dropout, false alleles, and null alleles, all of which are influenced by low DNA quality coupled with instances of genetic drift [97,99,100]. The presence of potential null alleles at all loci highlights the need to consider error rates in individual identification. Genotyping errors often result in significant deviations from Hardy-Weinberg equilibrium, particularly an excess of homozygotes [101] or an imbalance of genotype frequencies [102]. The complex social structure of elephants, often comprising female-offspring groups, may also bias P_(ID)_ estimate due to the presence of close relatives [103,104]. Evidence from Wilcoxon signed-rank tests and *M*-ratio analysis suggests a bottleneck event in the study population, potentially disrupting Hardy-Weinberg equilibrium and affecting P_(ID)_ estimates. Despite these factors, the observed P_(ID)_ and P_(ID)sibs_ values suggest that the data remain unbiased by close relatives [90,105].

This study demonstrated greater efficiency in individual identification than previous research using non-invasive samples and 10 loci in wild Thai elephants [106], likely due to the improved set of loci used. The microsatellite marker panels developed here show promise for non-invasive identification and are sufficient for future population studies aimed at conserving wild elephant populations.

## 5. Conclusions

This study provides a thorough genetic assessment of both captive and wild Asian elephant populations in Thailand, offering critical insights into their genetic diversity, individual identification, and population structure. Analyses using microsatellite loci and mtDNA D-loop sequencing revealed high levels of genetic diversity across all populations, despite some evidence of inbreeding. Three maternal haplogroups (α, β1, and a tentative β3) were identified, and substantial genetic variation was observed, particularly between the BCEP and MEP clusters, indicating distinct origins. Gene flow and introgression were detected between the NEI and EKS populations, likely as a result of individual or semen transfers, illustrating the influence of captive management practices on genetic structure. The presence of wild population traces within the captive gene pool reflects the historical capture and trade of elephants to support Thailand's tourism industry. Directional selective pressures were also identified in both wild and captive populations. The reduced panel of microsatellite markers proved to be highly efficient for individual identification and parentage verification, making it a practical tool for conservation efforts. Non-invasive sampling methods have shown strong potential for identifying individuals, providing valuable data for optimizing conservation programs. However, challenges surrounding non-invasive sampling methods have limited the use of microsatellites, which also demonstrate potential for individual identification. These findings contribute to the development of targeted strategies to safeguard the long-term survival of Asian elephants in Thailand. Further research should be conducted beyond Thailand to provide a more comprehensive understanding of these species’ status across Asia. Additionally, WGS/SNP panels could be used to increase genetic data resolution in order to provide broader insights into elephant population genomics across Asia.

## Supporting information

S1 Fig Plot based on Evanno’s ΔK showing patterns of population structure of 329 Asian elephant (Elephas maximus) individuals based on 18 microsatellite loci. (TIFF)

S2 Fig TreeMix analysis used to construct maximum likelihood trees for Thai Asian elephants (Elephas maximus): (a) Maximum likelihood tree and (b) residual fit evaluation for one migration event. The scale bar indicates ten times the average standard error of the values in the covariance matrix.
(TIFF)

S3 Fig Matching probability (MP), exclusion probability (PE), probability of identity (P(ID)), and probability of identity for siblings (P(ID)sibs) values for combinations of loci using 18 microsatellite loci for 329 Thai Asian elephants. Calculated using GenAlEx version 6.5.
(TIFF)

S4 Fig Matching probability (MP), exclusion probability (PE), probability of identity (P(ID)), and probability of identity for siblings (P(ID)sibs) values for combinations of six microsatellite loci selected via the ACO algorithm for 329 Thai Asian elephants. (TIFF)

S5 Fig Matching probability (MP), exclusion probability (PE), probability of identity (P(ID)), and probability of identity for siblings (P(ID)sibs) values for combinations of 18 microsatellite loci for nine fecal samples from Thai Asian elephants at Kui Buri National Park. Calculated using GenAlEx version 6.5.
(TIFF)

S6 Fig Phylogenetic tree based on mitochondrial DNA D-loop sequence data from Asian elephants (Elephas maximus) across five populations in this study. (TIFF)

S7 Fig Coalescent Bayesian skyline analysis output for all populations in this study. The black line represents the median estimated effective population size, while the blue areas indicate the 95% highest posterior density intervals.
(TIFF)

S1 Table Specimen populations of Asian elephants (*Elephas maximus*) in Thailand. All sequences have been deposited in the DNA Data Bank of Japan (DDBJ).
(DOCX)

S2 Table Microsatellite primers and sequences. (DOCX)

S3 Table Genetic diversity of 329 Asian elephants (*Elephas maximus*) based on 18 microsatellite loci. (DOCX)

S4 Table Distributions of *r*-values and *F*_IS_ values for Asian elephants (*Elephas maximus*). (DOCX)

S5 Table Pairwise genetic differentiation (*F*_ST_), pairwise *F*_ST_^ENA^ values with ENA correction for null alleles, and *R*_ST_ values for Asian elephants (*Elephas maximus*) based on eight microsatellite loci. The numbers indicate *p*-values, with 110 permutations.
(DOCX)

S6 Table Results of the analysis of molecular variance (AMOVA) for Asian elephants (*Elephas maximus*) based on 18 microsatellite loci. (DOCX)

S7 Table Source/recipient population comparisons showing mean migration rates and 95% confidence intervals, determined using BAYESASS software and microsatellite data from Asian elephants (*Elephas maximus*). (DOCX)

S8 Table Effective number of immigrants (*N*_m_) per generation (from population *i* to population *j*) for 329 Asian elephants (*Elephas maximus*) based on 18 microsatellite loci. (DOCX)

S9 Table Summary of microsatellite markers selected by the *PIC*+ACO algorithm according to various margin errors. (DOCX)

S10 Table Matching probability (MP), exclusion probability (PE), probability of identity (P_(ID)_), and probability of identity for siblings (P_(ID)sibs_) values per locus for 9 fecal samples from Thai Asian elephants at Kui Buri National Park, based on 18 microsatellite loci. (DOCX)
