# Supplementary figures and images for: Genetic insights for enhancing conservation strategies in captive and wild Asian elephants through improved non-invasive DNA-based individual identification
Source: PLoS One. 2025 May 12;20(5):e0320480. doi: 10.1371/journal.pone.0320480 (PMC12068619; doi:10.1371/journal.pone.0320480)

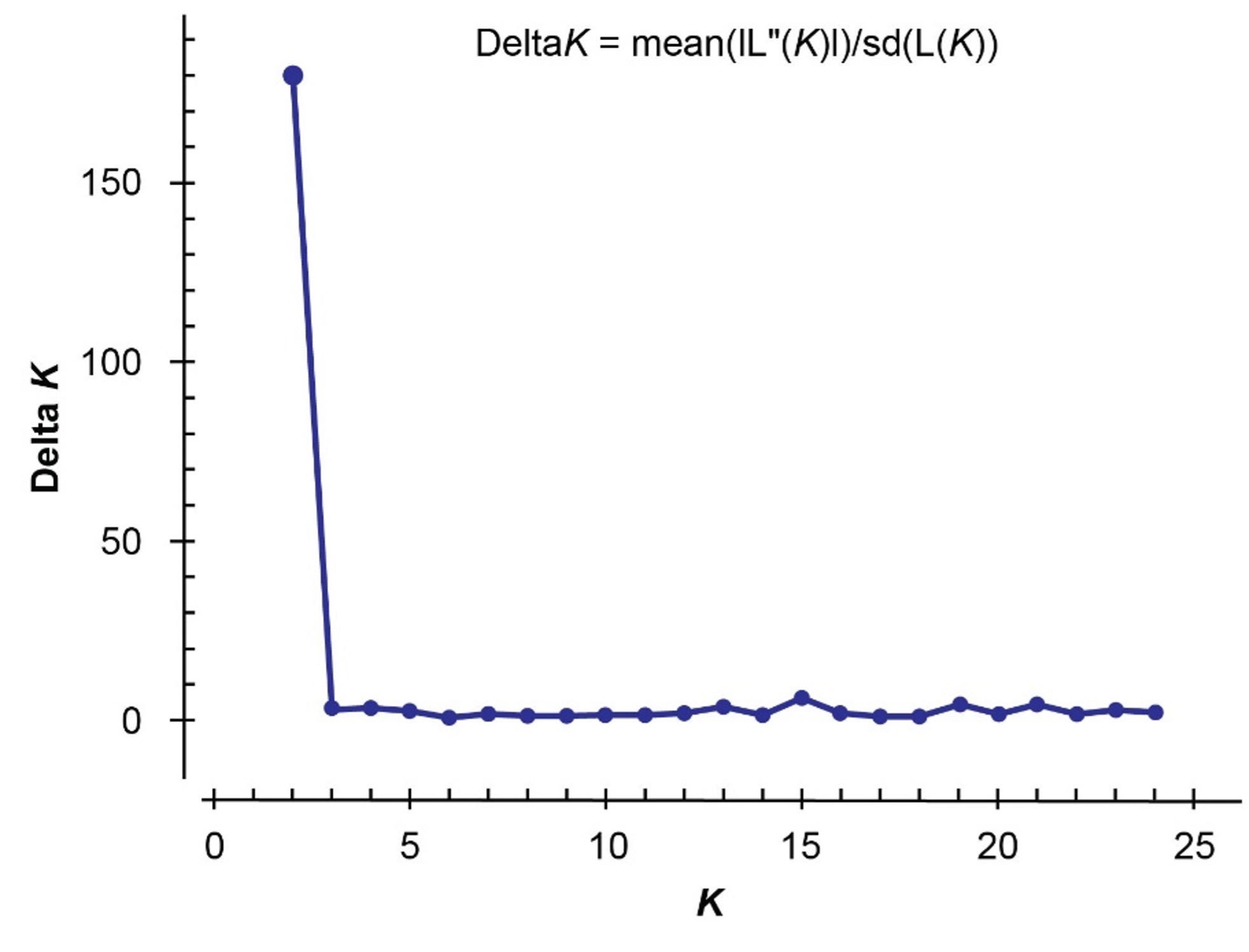

Supplement: S1 Fig — (TIFF) [file pone.0320480.s001.tiff]

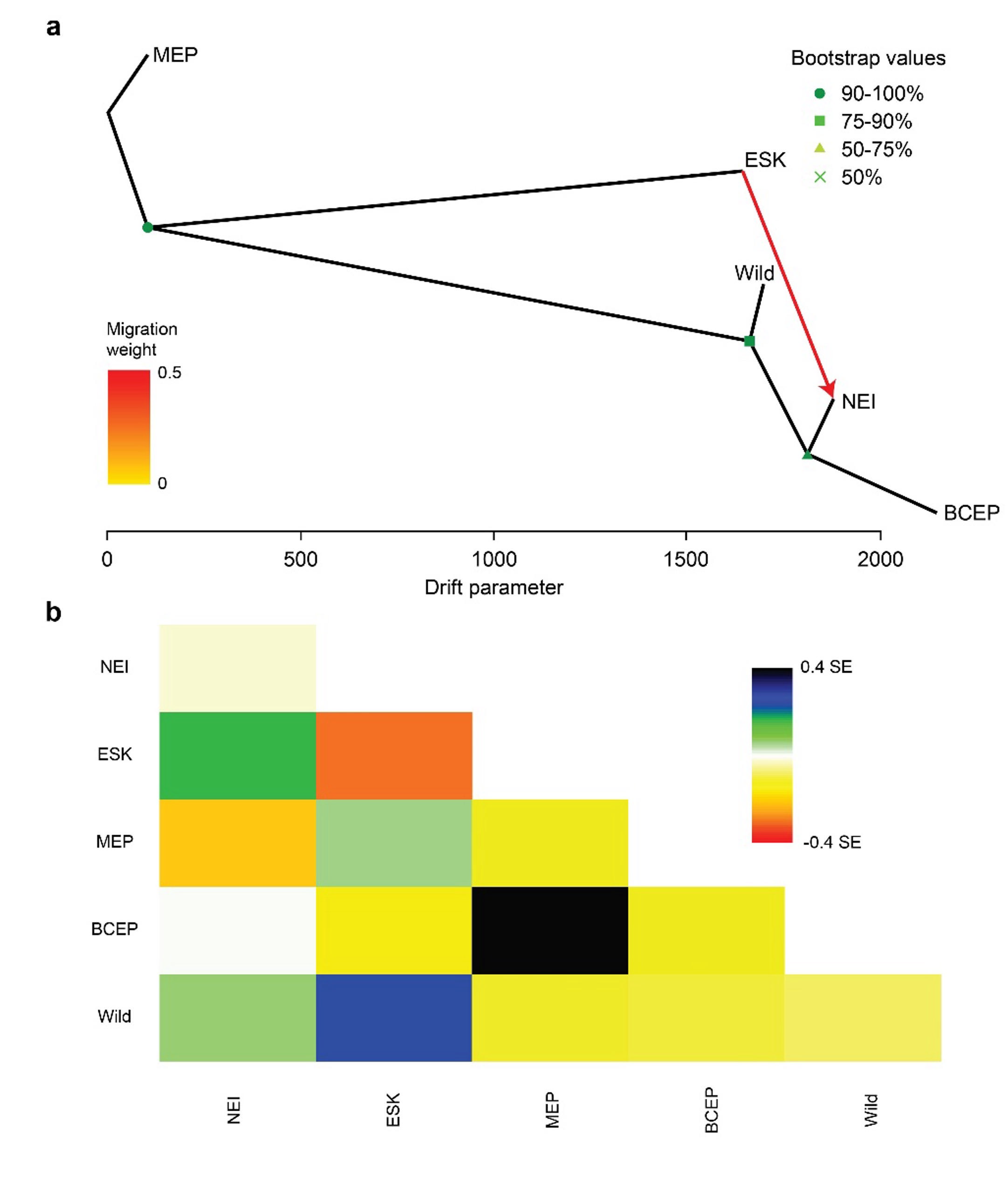

Supplement: S2 Fig — The scale bar indicates ten times the average standard error of the values in the covariance matrix. (TIFF) [file pone.0320480.s002.tiff]

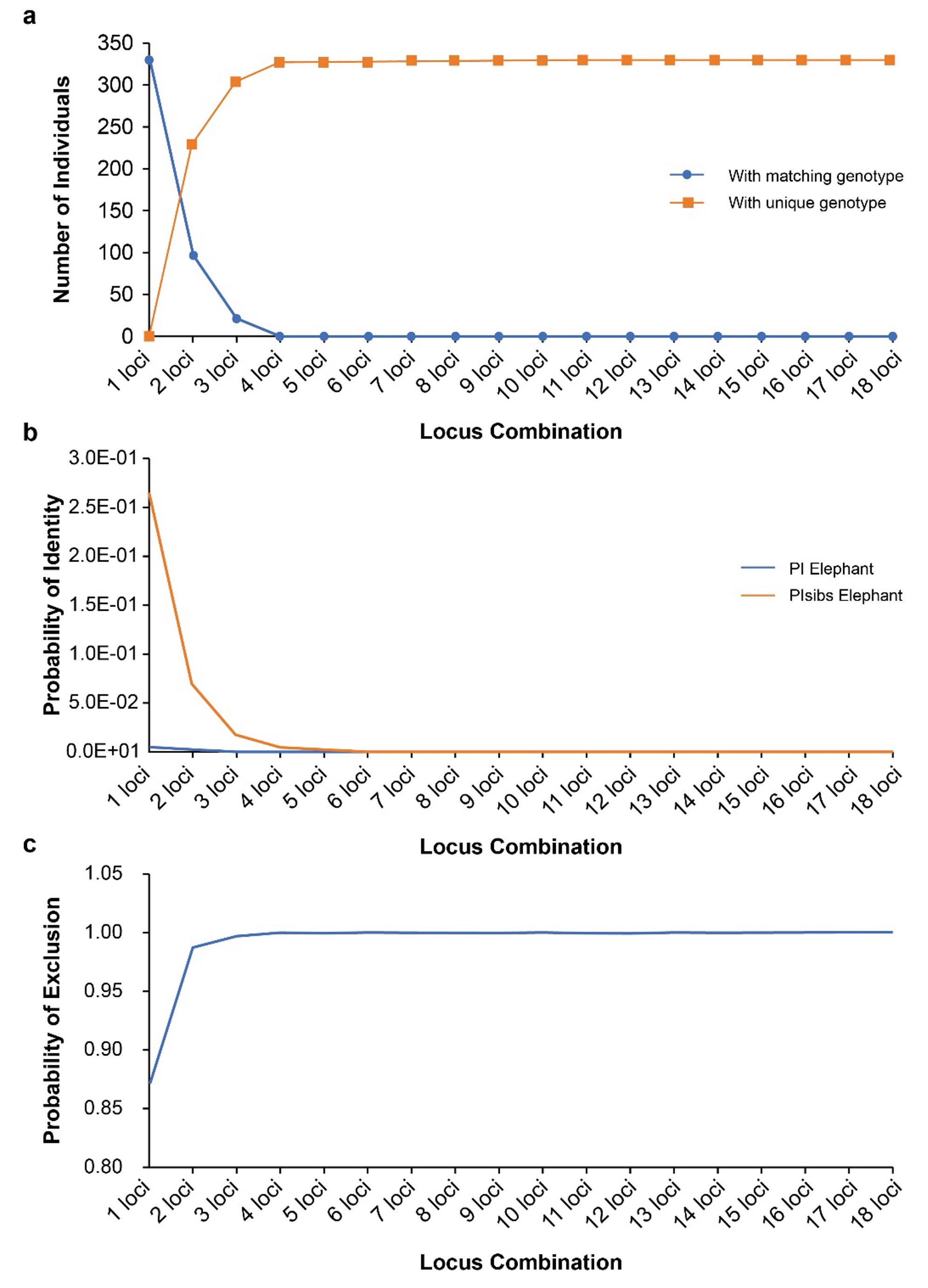

Supplement: S3 Fig — Calculated using GenAlEx version 6.5. (TIFF) [file pone.0320480.s003.tiff]

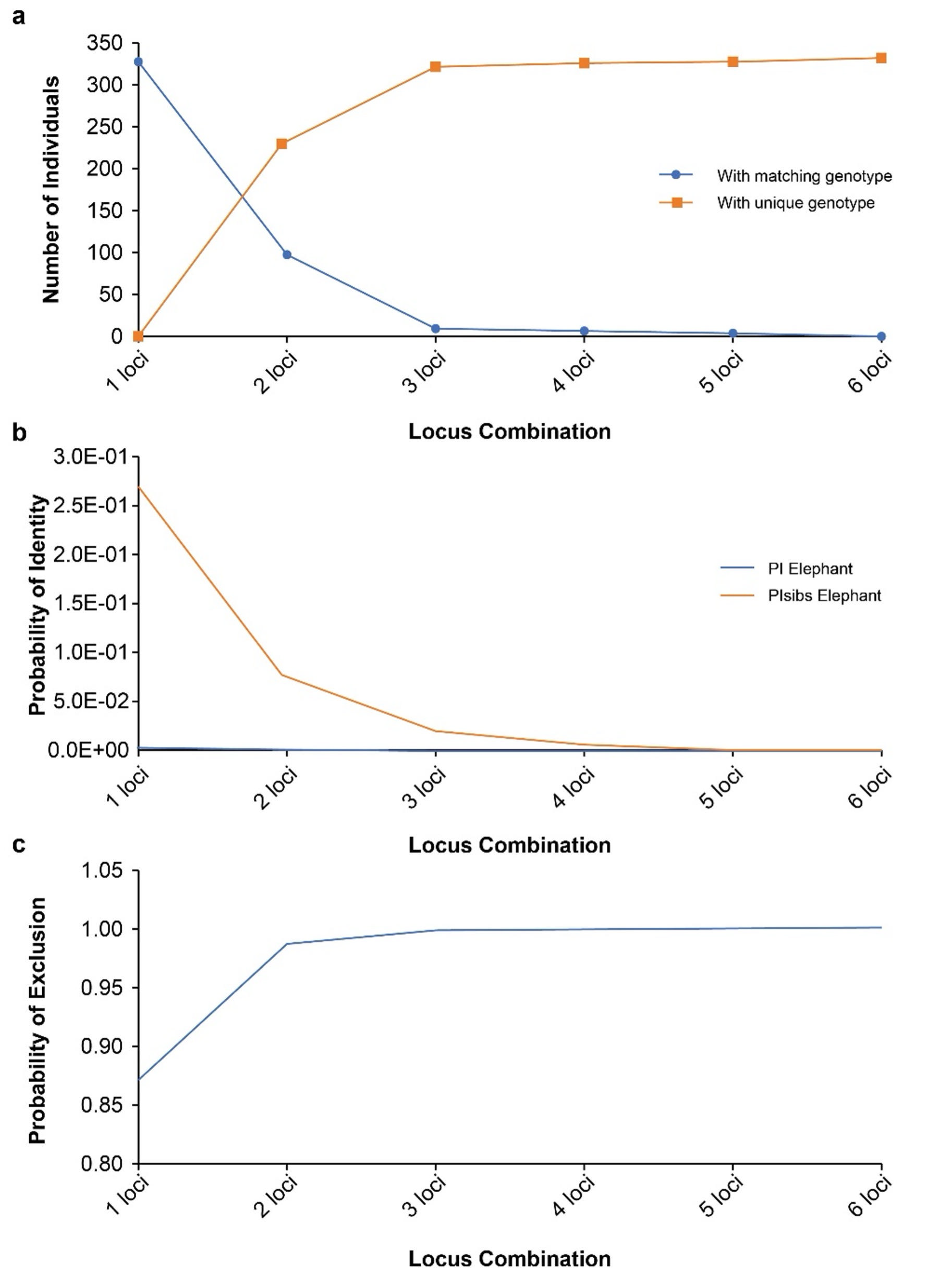

Supplement: S4 Fig — (TIFF) [file pone.0320480.s004.tiff]

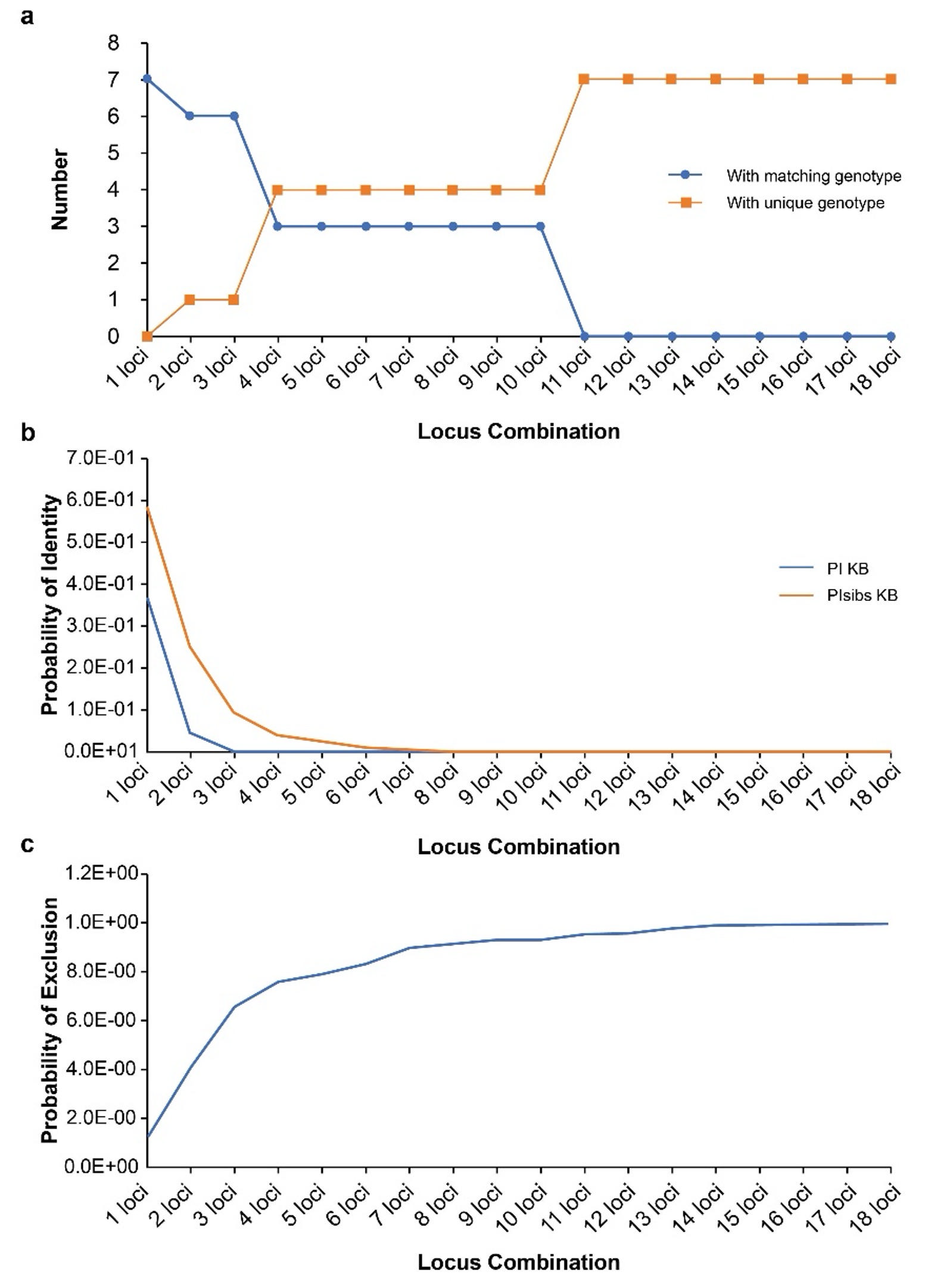

Supplement: S5 Fig — Calculated using GenAlEx version 6.5. (TIFF) [file pone.0320480.s005.tiff]

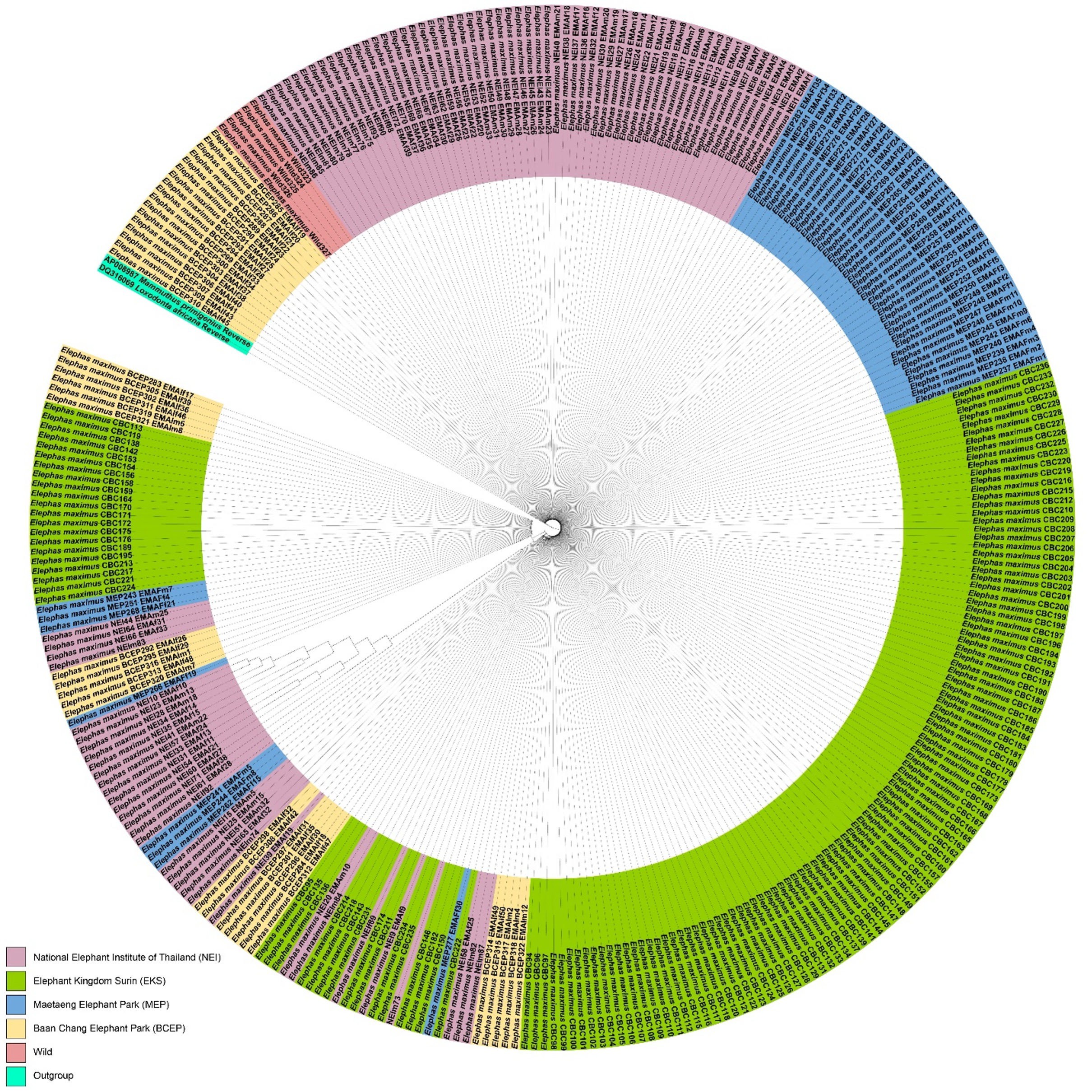

Supplement: S6 Fig — (TIFF) [file pone.0320480.s006.tiff]

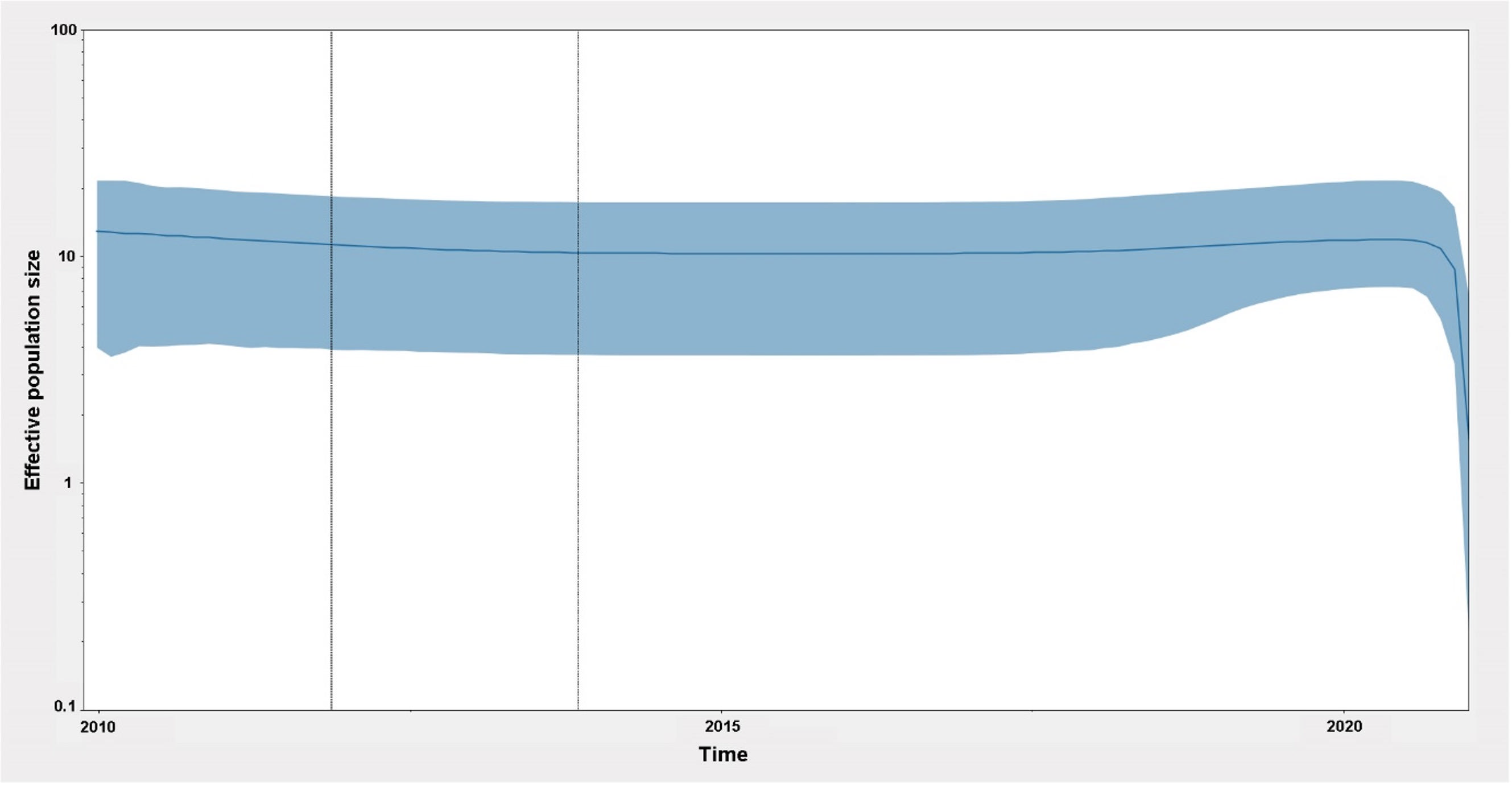

Supplement: S7 Fig — The black line represents the median estimated effective population size, while the blue areas indicate the 95% highest posterior density intervals. (TIFF) [file pone.0320480.s007.tiff]
