# Supplementary material for: Genetic insights for enhancing conservation strategies in captive and wild Asian elephants through improved non-invasive DNA-based individual identification
Source: PLoS One. 2025 May 12;20(5):e0320480. doi: 10.1371/journal.pone.0320480 (PMC12068619; doi:10.1371/journal.pone.0320480)
Supplement: S1 Table — All sequences have been deposited in the DNA Data Bank of Japan (DDBJ). (DOCX) [file pone.0320480.s008.docx]

S1 Table. Specimen populations of Asian elephants (*Elephas maximus*) in Thailand. All sequences were deposited in the DNA Data Bank of Japan (DDBJ)

| **No.** | **Abbreviation/Code** | **Coordinate** | **Locality** | **Mitochondrial D loop DDBJ accession number** |
| --- | --- | --- | --- | --- |
|  | NEI1 | 18°21'35.7"N 99°14'49.9"E | National Elephant Institute of Thailand | LC699890 |
|  | NEI2 | 18°21'35.7"N 99°14'49.9"E | National Elephant Institute of Thailand, Lumpang | LC699891 |
|  | NEI3 | 18°21'35.7"N 99°14'49.9"E | National Elephant Institute of Thailand, Lumpang | LC699892 |
|  | NEI4 | 18°21'35.7"N 99°14'49.9"E | National Elephant Institute of Thailand, Lumpang | LC699893 |
|  | NEI5 | 18°21'35.7"N 99°14'49.9"E | National Elephant Institute of Thailand, Lumpang | LC699894 |
|  | NEI6 | 18°21'35.7"N 99°14'49.9"E | National Elephant Institute of Thailand, Lumpang | LC699895 |
|  | NEI7 | 18°21'35.7"N 99°14'49.9"E | National Elephant Institute of Thailand, Lumpang | LC699896 |
|  | NEI8 | 18°21'35.7"N 99°14'49.9"E | National Elephant Institute of Thailand, Lumpang | LC699897 |
|  | NEI9 | 18°21'35.7"N 99°14'49.9"E | National Elephant Institute of Thailand, Lumpang | LC699898 |
|  | NEI10 | 18°21'35.7"N 99°14'49.9"E | National Elephant Institute of Thailand, Lumpang | LC699899 |
|  | NEI11 | 18°21'35.7"N 99°14'49.9"E | National Elephant Institute of Thailand, Lumpang | LC699900 |
|  | NEI12 | 18°21'35.7"N 99°14'49.9"E | National Elephant Institute of Thailand, Lumpang | LC699901 |
|  | NEI13 | 18°21'35.7"N 99°14'49.9"E | National Elephant Institute of Thailand, Lumpang | LC699902 |
|  | NEI14 | 18°21'35.7"N 99°14'49.9"E | National Elephant Institute of Thailand, Lumpang | LC699903 |
|  | NEI15 | 18°21'35.7"N 99°14'49.9"E | National Elephant Institute of Thailand, Lumpang | LC699904 |
|  | NEI16 | 18°21'35.7"N 99°14'49.9"E | National Elephant Institute of Thailand, Lumpang | LC699905 |
|  | NEI17 | 18°21'35.7"N 99°14'49.9"E | National Elephant Institute of Thailand, Lumpang | LC699906 |
|  | NEI18 | 18°21'35.7"N 99°14'49.9"E | National Elephant Institute of Thailand, Lumpang | LC699907 |
|  | NEI19 | 18°21'35.7"N 99°14'49.9"E | National Elephant Institute of Thailand, Lumpang | LC699908 |
|  | NEI20 | 18°21'35.7"N 99°14'49.9"E | National Elephant Institute of Thailand, Lumpang | LC699909 |
|  | NEI21 | 18°21'35.7"N 99°14'49.9"E | National Elephant Institute of Thailand, Lumpang | LC699910 |
|  | NEI22 | 18°21'35.7"N 99°14'49.9"E | National Elephant Institute of Thailand, Lumpang | LC699911 |
|  | NEI23 | 18°21'35.7"N 99°14'49.9"E | National Elephant Institute of Thailand, Lumpang | Goodmorinig |
|  | NEI24 | 18°21'35.7"N 99°14'49.9"E | National Elephant Institute of Thailand, Lumpang | LC699913 |
|  | NEI25 | 18°21'35.7"N 99°14'49.9"E | National Elephant Institute of Thailand, Lumpang | LC699914 |
|  | NEI26 | 18°21'35.7"N 99°14'49.9"E | National Elephant Institute of Thailand, Lumpang | LC699915 |
|  | NEI27 | 18°21'35.7"N 99°14'49.9"E | National Elephant Institute of Thailand, Lumpang | LC699916 |
|  | NEI28 | 18°21'35.7"N 99°14'49.9"E | National Elephant Institute of Thailand, Lumpang | LC699917 |
|  | NEI29 | 18°21'35.7"N 99°14'49.9"E | National Elephant Institute of Thailand, Lumpang | LC699918 |
|  | NEI30 | 18°21'35.7"N 99°14'49.9"E | National Elephant Institute of Thailand, Lumpang | LC699919 |
|  | NEI31 | 18°21'35.7"N 99°14'49.9"E | National Elephant Institute of Thailand, Lumpang | LC699920 |
|  | NEI32 | 18°21'35.7"N 99°14'49.9"E | National Elephant Institute of Thailand, Lumpang | LC699921 |
|  | NEI33 | 18°21'35.7"N 99°14'49.9"E | National Elephant Institute of Thailand, Lumpang | LC699922 |
|  | NEI34 | 18°21'35.7"N 99°14'49.9"E | National Elephant Institute of Thailand, Lumpang | LC699923 |
|  | NEI35 | 18°21'35.7"N 99°14'49.9"E | National Elephant Institute of Thailand, Lumpang | LC699924 |
|  | NEI36 | 18°21'35.7"N 99°14'49.9"E | National Elephant Institute of Thailand, Lumpang | LC699925 |
|  | NEI37 | 18°21'35.7"N 99°14'49.9"E | National Elephant Institute of Thailand, Lumpang | LC699926 |
|  | NEI38 | 18°21'35.7"N 99°14'49.9"E | National Elephant Institute of Thailand, Lumpang | LC699927 |
|  | NEI39 | 18°21'35.7"N 99°14'49.9"E | National Elephant Institute of Thailand, Lumpang | LC699928 |
|  | NEI40 | 18°21'35.7"N 99°14'49.9"E | National Elephant Institute of Thailand, Lumpang | LC699929 |
|  | NEI41 | 18°21'35.7"N 99°14'49.9"E | National Elephant Institute of Thailand, Lumpang | LC699930 |
|  | NEI42 | 18°21'35.7"N 99°14'49.9"E | National Elephant Institute of Thailand, Lumpang | LC699931 |
|  | NEI43 | 18°21'35.7"N 99°14'49.9"E | National Elephant Institute of Thailand, Lumpang | LC699932 |
|  | NEI44 | 18°21'35.7"N 99°14'49.9"E | National Elephant Institute of Thailand, Lumpang | LC699933 |
|  | NEI45 | 18°21'35.7"N 99°14'49.9"E | National Elephant Institute of Thailand, Lumpang | LC699934 |
|  | NEI46 | 18°21'35.7"N 99°14'49.9"E | National Elephant Institute of Thailand, Lumpang | LC699935 |
|  | NEI47 | 18°21'35.7"N 99°14'49.9"E | National Elephant Institute of Thailand, Lumpang | LC699936 |
|  | NEI48 | 18°21'35.7"N 99°14'49.9"E | National Elephant Institute of Thailand, Lumpang | LC699937 |
|  | NEI49 | 18°21'35.7"N 99°14'49.9"E | National Elephant Institute of Thailand, Lumpang | LC699938 |
|  | NEI50 | 18°21'35.7"N 99°14'49.9"E | National Elephant Institute of Thailand, Lumpang | LC699939 |
|  | NEI51 | 18°21'35.7"N 99°14'49.9"E | National Elephant Institute of Thailand, Lumpang | LC699940 |
|  | NEI52 | 18°21'35.7"N 99°14'49.9"E | National Elephant Institute of Thailand, Lumpang | LC699941 |
|  | NEI53 | 18°21'35.7"N 99°14'49.9"E | National Elephant Institute of Thailand, Lumpang | LC699942 |
|  | NEI54 | 18°21'35.7"N 99°14'49.9"E | National Elephant Institute of Thailand, Lumpang | LC699943 |
|  | NEI55 | 18°21'35.7"N 99°14'49.9"E | National Elephant Institute of Thailand, Lumpang | LC699944 |
|  | NEI56 | 18°21'35.7"N 99°14'49.9"E | National Elephant Institute of Thailand, Lumpang | LC699945 |
|  | NEI57 | 18°21'35.7"N 99°14'49.9"E | National Elephant Institute of Thailand, Lumpang | LC699946 |
|  | NEI58 | 18°21'35.7"N 99°14'49.9"E | National Elephant Institute of Thailand, Lumpang | LC699947 |
|  | NEI59 | 18°21'35.7"N 99°14'49.9"E | National Elephant Institute of Thailand, Lumpang | LC699948 |
|  | NEI60 | 18°21'35.7"N 99°14'49.9"E | National Elephant Institute of Thailand, Lumpang | LC699949 |
|  | NEI61 | 18°21'35.7"N 99°14'49.9"E | National Elephant Institute of Thailand, Lumpang | LC699950 |
|  | NEI62 | 18°21'35.7"N 99°14'49.9"E | National Elephant Institute of Thailand, Lumpang | LC699951 |
|  | NEI63 | 18°21'35.7"N 99°14'49.9"E | National Elephant Institute of Thailand, Lumpang | LC699952 |
|  | NEI64 | 18°21'35.7"N 99°14'49.9"E | National Elephant Institute of Thailand, Lumpang | LC699953 |
|  | NEI65 | 18°21'35.7"N 99°14'49.9"E | National Elephant Institute of Thailand, Lumpang | LC699954 |
|  | NEI66 | 18°21'35.7"N 99°14'49.9"E | National Elephant Institute of Thailand, Lumpang | LC699955 |
|  | NEI67 | 18°21'35.7"N 99°14'49.9"E | National Elephant Institute of Thailand, Lumpang | LC699956 |
|  | NEI68 | 18°21'35.7"N 99°14'49.9"E | National Elephant Institute of Thailand, Lumpang | LC699957 |
|  | NEI69 | 18°21'35.7"N 99°14'49.9"E | National Elephant Institute of Thailand, Lumpang | LC699958 |
|  | NEI70 | 18°21'35.7"N 99°14'49.9"E | National Elephant Institute of Thailand, Lumpang | LC699959 |
|  | NEI71 | 18°21'35.7"N 99°14'49.9"E | National Elephant Institute of Thailand, Lumpang | LC699960 |
|  | NEI72 | 18°21'35.7"N 99°14'49.9"E | National Elephant Institute of Thailand, Lumpang | LC699961 |
|  | NEIm73 | 18°21'35.7"N 99°14'49.9"E | National Elephant Institute of Thailand, Lumpang | LC789907 |
|  | NEIm74 | 18°21'35.7"N 99°14'49.9"E | National Elephant Institute of Thailand, Lumpang | LC789908 |
|  | NEIm75 | 18°21'35.7"N 99°14'49.9"E | National Elephant Institute of Thailand, Lumpang | LC789909 |
|  | NEIm76 | 18°21'35.7"N 99°14'49.9"E | National Elephant Institute of Thailand, Lumpang | LC789910 |
|  | NEIm77 | 18°21'35.7"N 99°14'49.9"E | National Elephant Institute of Thailand, Lumpang | LC789911 |
|  | NEIm78 | 18°21'35.7"N 99°14'49.9"E | National Elephant Institute of Thailand, Lumpang | LC789912 |
|  | NEIm79 | 18°21'35.7"N 99°14'49.9"E | National Elephant Institute of Thailand, Lumpang | LC789913 |
|  | NEIm80 | 18°21'35.7"N 99°14'49.9"E | National Elephant Institute of Thailand, Lumpang | LC789914 |
|  | NEIm81 | 18°21'35.7"N 99°14'49.9"E | National Elephant Institute of Thailand, Lumpang | LC789915 |
|  | NEIm82 | 18°21'35.7"N 99°14'49.9"E | National Elephant Institute of Thailand, Lumpang | LC789916 |
|  | NEIm83 | 18°21'35.7"N 99°14'49.9"E | National Elephant Institute of Thailand, Lumpang | LC789917 |
|  | NEIm84 | 18°21'35.7"N 99°14'49.9"E | National Elephant Institute of Thailand, Lumpang | LC789918 |
|  | NEIm85 | 18°21'35.7"N 99°14'49.9"E | National Elephant Institute of Thailand, Lumpang | LC789919 |
|  | NEIm86 | 18°21'35.7"N 99°14'49.9"E | National Elephant Institute of Thailand, Lumpang | LC789920 |
|  | NEIm87 | 18°21'35.7"N 99°14'49.9"E | National Elephant Institute of Thailand, Lumpang | LC789921 |
|  | NEIf88 | 18°21'35.7"N 99°14'49.9"E | National Elephant Institute of Thailand, Lumpang | LC789901 |
|  | NEIf89 | 18°21'35.7"N 99°14'49.9"E | National Elephant Institute of Thailand, Lumpang | LC789902 |
|  | NEIf90 | 18°21'35.7"N 99°14'49.9"E | National Elephant Institute of Thailand, Lumpang | LC789903 |
|  | NEIf91 | 18°21'35.7"N 99°14'49.9"E | National Elephant Institute of Thailand, Lumpang | LC789904 |
|  | NEIf92 | 18°21'35.7"N 99°14'49.9"E | National Elephant Institute of Thailand, Lumpang | LC789905 |
|  | NEIf93 | 18°21'35.7"N 99°14'49.9"E | National Elephant Institute of Thailand, Lumpang | LC789906 |
|  | CBC94 | 14°59'28.9"N 103°36'25.5"E | Elephant Kingdom Surin, Surin | LC789761 |
|  | CBC95 | 14°59'28.9"N 103°36'25.5"E | Elephant Kingdom Surin, Surin | LC789762 |
|  | CBC96 | 14°59'28.9"N 103°36'25.5"E | Elephant Kingdom Surin, Surin | LC789763 |
|  | CBC97 | 14°59'28.9"N 103°36'25.5"E | Elephant Kingdom Surin, Surin | LC789764 |
|  | CBC98 | 14°59'28.9"N 103°36'25.5"E | Elephant Kingdom Surin, Surin | LC789765 |
|  | CBC99 | 14°59'28.9"N 103°36'25.5"E | Elephant Kingdom Surin, Surin | LC789766 |
|  | CBC100 | 14°59'28.9"N 103°36'25.5"E | Elephant Kingdom Surin, Surin | LC789767 |
|  | CBC101 | 14°59'28.9"N 103°36'25.5"E | Elephant Kingdom Surin, Surin | LC789768 |
|  | CBC102 | 14°59'28.9"N 103°36'25.5"E | Elephant Kingdom Surin, Surin | LC789769 |
|  | CBC103 | 14°59'28.9"N 103°36'25.5"E | Elephant Kingdom Surin, Surin | LC789770 |
|  | CBC104 | 14°59'28.9"N 103°36'25.5"E | Elephant Kingdom Surin, Surin | LC789771 |
|  | CBC105 | 14°59'28.9"N 103°36'25.5"E | Elephant Kingdom Surin, Surin | LC789772 |
|  | CBC106 | 14°59'28.9"N 103°36'25.5"E | Elephant Kingdom Surin, Surin | LC789773 |
|  | CBC107 | 14°59'28.9"N 103°36'25.5"E | Elephant Kingdom Surin, Surin | LC789774 |
|  | CBC108 | 14°59'28.9"N 103°36'25.5"E | Elephant Kingdom Surin, Surin | LC789775 |
|  | CBC109 | 14°59'28.9"N 103°36'25.5"E | Elephant Kingdom Surin, Surin | LC789776 |
|  | CBC110 | 14°59'28.9"N 103°36'25.5"E | Elephant Kingdom Surin, Surin | LC789777 |
|  | CBC111 | 14°59'28.9"N 103°36'25.5"E | Elephant Kingdom Surin, Surin | LC789778 |
|  | CBC112 | 14°59'28.9"N 103°36'25.5"E | Elephant Kingdom Surin, Surin | LC789779 |
|  | CBC113 | 14°59'28.9"N 103°36'25.5"E | Elephant Kingdom Surin, Surin | LC789780 |
|  | CBC114 | 14°59'28.9"N 103°36'25.5"E | Elephant Kingdom Surin, Surin | LC789781 |
|  | CBC115 | 14°59'28.9"N 103°36'25.5"E | Elephant Kingdom Surin, Surin | LC789782 |
|  | CBC116 | 14°59'28.9"N 103°36'25.5"E | Elephant Kingdom Surin, Surin | LC789783 |
|  | CBC117 | 14°59'28.9"N 103°36'25.5"E | Elephant Kingdom Surin, Surin | LC789784 |
|  | CBC118 | 14°59'28.9"N 103°36'25.5"E | Elephant Kingdom Surin, Surin | LC789785 |
|  | CBC119 | 14°59'28.9"N 103°36'25.5"E | Elephant Kingdom Surin, Surin | LC789786 |
|  | CBC120 | 14°59'28.9"N 103°36'25.5"E | Elephant Kingdom Surin, Surin | LC789787 |
|  | CBC121 | 14°59'28.9"N 103°36'25.5"E | Elephant Kingdom Surin, Surin | LC789788 |
|  | CBC122 | 14°59'28.9"N 103°36'25.5"E | Elephant Kingdom Surin, Surin | LC789789 |
|  | CBC123 | 14°59'28.9"N 103°36'25.5"E | Elephant Kingdom Surin, Surin | LC789790 |
|  | CBC124 | 14°59'28.9"N 103°36'25.5"E | Elephant Kingdom Surin, Surin | LC789791 |
|  | CBC125 | 14°59'28.9"N 103°36'25.5"E | Elephant Kingdom Surin, Surin | LC789792 |
|  | CBC126 | 14°59'28.9"N 103°36'25.5"E | Elephant Kingdom Surin, Surin | LC789793 |
|  | CBC127 | 14°59'28.9"N 103°36'25.5"E | Elephant Kingdom Surin, Surin | LC789794 |
|  | CBC128 | 14°59'28.9"N 103°36'25.5"E | Elephant Kingdom Surin, Surin | LC789795 |
|  | CBC129 | 14°59'28.9"N 103°36'25.5"E | Elephant Kingdom Surin, Surin | LC789796 |
|  | CBC130 | 14°59'28.9"N 103°36'25.5"E | Elephant Kingdom Surin, Surin | LC789797 |
|  | CBC131 | 14°59'28.9"N 103°36'25.5"E | Elephant Kingdom Surin, Surin | LC789798 |
|  | CBC132 | 14°59'28.9"N 103°36'25.5"E | Elephant Kingdom Surin, Surin | LC789799 |
|  | CBC133 | 14°59'28.9"N 103°36'25.5"E | Elephant Kingdom Surin, Surin | LC789800 |
|  | CBC134 | 14°59'28.9"N 103°36'25.5"E | Elephant Kingdom Surin, Surin | LC789801 |
|  | CBC135 | 14°59'28.9"N 103°36'25.5"E | Elephant Kingdom Surin, Surin | LC789802 |
|  | CBC136 | 14°59'28.9"N 103°36'25.5"E | Elephant Kingdom Surin, Surin | LC789803 |
|  | CBC137 | 14°59'28.9"N 103°36'25.5"E | Elephant Kingdom Surin, Surin | LC789804 |
|  | CBC138 | 14°59'28.9"N 103°36'25.5"E | Elephant Kingdom Surin, Surin | LC789805 |
|  | CBC139 | 14°59'28.9"N 103°36'25.5"E | Elephant Kingdom Surin, Surin | LC789806 |
|  | CBC140 | 14°59'28.9"N 103°36'25.5"E | Elephant Kingdom Surin, Surin | LC789807 |
|  | CBC141 | 14°59'28.9"N 103°36'25.5"E | Elephant Kingdom Surin, Surin | LC789808 |
|  | CBC142 | 14°59'28.9"N 103°36'25.5"E | Elephant Kingdom Surin, Surin | LC789809 |
|  | CBC143 | 14°59'28.9"N 103°36'25.5"E | Elephant Kingdom Surin, Surin | LC789810 |
|  | CBC144 | 14°59'28.9"N 103°36'25.5"E | Elephant Kingdom Surin, Surin | LC789811 |
|  | CBC145 | 14°59'28.9"N 103°36'25.5"E | Elephant Kingdom Surin, Surin | LC789812 |
|  | CBC146 | 14°59'28.9"N 103°36'25.5"E | Elephant Kingdom Surin, Surin | LC789813 |
|  | CBC147 | 14°59'28.9"N 103°36'25.5"E | Elephant Kingdom Surin, Surin | LC789814 |
|  | CBC148 | 14°59'28.9"N 103°36'25.5"E | Elephant Kingdom Surin, Surin | LC789815 |
|  | CBC149 | 14°59'28.9"N 103°36'25.5"E | Elephant Kingdom Surin, Surin | LC789816 |
|  | CBC150 | 14°59'28.9"N 103°36'25.5"E | Elephant Kingdom Surin, Surin | LC789817 |
|  | CBC151 | 14°59'28.9"N 103°36'25.5"E | Elephant Kingdom Surin, Surin | LC789818 |
|  | CBC152 | 14°59'28.9"N 103°36'25.5"E | Elephant Kingdom Surin, Surin | LC789819 |
|  | CBC153 | 14°59'28.9"N 103°36'25.5"E | Elephant Kingdom Surin, Surin | LC789820 |
|  | CBC154 | 14°59'28.9"N 103°36'25.5"E | Elephant Kingdom Surin, Surin | LC789821 |
|  | CBC155 | 14°59'28.9"N 103°36'25.5"E | Elephant Kingdom Surin, Surin | LC789822 |
|  | CBC156 | 14°59'28.9"N 103°36'25.5"E | Elephant Kingdom Surin, Surin | LC789823 |
|  | CBC157 | 14°59'28.9"N 103°36'25.5"E | Elephant Kingdom Surin, Surin | LC789824 |
|  | CBC158 | 14°59'28.9"N 103°36'25.5"E | Elephant Kingdom Surin, Surin | LC789825 |
|  | CBC159 | 14°59'28.9"N 103°36'25.5"E | Elephant Kingdom Surin, Surin | LC789826 |
|  | CBC160 | 14°59'28.9"N 103°36'25.5"E | Elephant Kingdom Surin, Surin | LC789827 |
|  | CBC161 | 14°59'28.9"N 103°36'25.5"E | Elephant Kingdom Surin, Surin | LC789828 |
|  | CBC162 | 14°59'28.9"N 103°36'25.5"E | Elephant Kingdom Surin, Surin | LC789829 |
|  | CBC163 | 14°59'28.9"N 103°36'25.5"E | Elephant Kingdom Surin, Surin | LC789830 |
|  | CBC164 | 14°59'28.9"N 103°36'25.5"E | Elephant Kingdom Surin, Surin | LC789831 |
|  | CBC165 | 14°59'28.9"N 103°36'25.5"E | Elephant Kingdom Surin, Surin | LC789832 |
|  | CBC166 | 14°59'28.9"N 103°36'25.5"E | Elephant Kingdom Surin, Surin | LC789833 |
|  | CBC167 | 14°59'28.9"N 103°36'25.5"E | Elephant Kingdom Surin, Surin | LC789834 |
|  | CBC168 | 14°59'28.9"N 103°36'25.5"E | Elephant Kingdom Surin, Surin | LC789835 |
|  | CBC169 | 14°59'28.9"N 103°36'25.5"E | Elephant Kingdom Surin, Surin | LC789836 |
|  | CBC170 | 14°59'28.9"N 103°36'25.5"E | Elephant Kingdom Surin, Surin | LC789837 |
|  | CBC171 | 14°59'28.9"N 103°36'25.5"E | Elephant Kingdom Surin, Surin | LC789838 |
|  | CBC172 | 14°59'28.9"N 103°36'25.5"E | Elephant Kingdom Surin, Surin | LC789839 |
|  | CBC173 | 14°59'28.9"N 103°36'25.5"E | Elephant Kingdom Surin, Surin | LC789840 |
|  | CBC174 | 14°59'28.9"N 103°36'25.5"E | Elephant Kingdom Surin, Surin | LC789841 |
|  | CBC175 | 14°59'28.9"N 103°36'25.5"E | Elephant Kingdom Surin, Surin | LC789842 |
|  | CBC176 | 14°59'28.9"N 103°36'25.5"E | Elephant Kingdom Surin, Surin | LC789843 |
|  | CBC177 | 14°59'28.9"N 103°36'25.5"E | Elephant Kingdom Surin, Surin | LC789844 |
|  | CBC178 | 14°59'28.9"N 103°36'25.5"E | Elephant Kingdom Surin, Surin | LC789845 |
|  | CBC179 | 14°59'28.9"N 103°36'25.5"E | Elephant Kingdom Surin, Surin | LC789846 |
|  | CBC180 | 14°59'28.9"N 103°36'25.5"E | Elephant Kingdom Surin, Surin | LC789847 |
|  | CBC181 | 14°59'28.9"N 103°36'25.5"E | Elephant Kingdom Surin, Surin | LC789848 |
|  | CBC182 | 14°59'28.9"N 103°36'25.5"E | Elephant Kingdom Surin, Surin | LC789849 |
|  | CBC183 | 14°59'28.9"N 103°36'25.5"E | Elephant Kingdom Surin, Surin | LC789850 |
|  | CBC184 | 14°59'28.9"N 103°36'25.5"E | Elephant Kingdom Surin, Surin | LC789851 |
|  | CBC185 | 14°59'28.9"N 103°36'25.5"E | Elephant Kingdom Surin, Surin | LC789852 |
|  | CBC186 | 14°59'28.9"N 103°36'25.5"E | Elephant Kingdom Surin, Surin | LC789853 |
|  | CBC187 | 14°59'28.9"N 103°36'25.5"E | Elephant Kingdom Surin, Surin | LC789854 |
|  | CBC188 | 14°59'28.9"N 103°36'25.5"E | Elephant Kingdom Surin, Surin | LC789855 |
|  | CBC189 | 14°59'28.9"N 103°36'25.5"E | Elephant Kingdom Surin, Surin | LC789856 |
|  | CBC190 | 14°59'28.9"N 103°36'25.5"E | Elephant Kingdom Surin, Surin | LC789857 |
|  | CBC191 | 14°59'28.9"N 103°36'25.5"E | Elephant Kingdom Surin, Surin | LC789858 |
|  | CBC192 | 14°59'28.9"N 103°36'25.5"E | Elephant Kingdom Surin, Surin | LC789859 |
|  | CBC193 | 14°59'28.9"N 103°36'25.5"E | Elephant Kingdom Surin, Surin | LC789860 |
|  | CBC194 | 14°59'28.9"N 103°36'25.5"E | Elephant Kingdom Surin, Surin | LC789861 |
|  | CBC195 | 14°59'28.9"N 103°36'25.5"E | Elephant Kingdom Surin, Surin | LC789862 |
|  | CBC196 | 14°59'28.9"N 103°36'25.5"E | Elephant Kingdom Surin, Surin | LC789863 |
|  | CBC197 | 14°59'28.9"N 103°36'25.5"E | Elephant Kingdom Surin, Surin | LC789864 |
|  | CBC198 | 14°59'28.9"N 103°36'25.5"E | Elephant Kingdom Surin, Surin | LC789865 |
|  | CBC199 | 14°59'28.9"N 103°36'25.5"E | Elephant Kingdom Surin, Surin | LC789866 |
|  | CBC200 | 14°59'28.9"N 103°36'25.5"E | Elephant Kingdom Surin, Surin | LC789867 |
|  | CBC201 | 14°59'28.9"N 103°36'25.5"E | Elephant Kingdom Surin, Surin | LC789868 |
|  | CBC202 | 14°59'28.9"N 103°36'25.5"E | Elephant Kingdom Surin, Surin | LC789869 |
|  | CBC203 | 14°59'28.9"N 103°36'25.5"E | Elephant Kingdom Surin, Surin | LC789870 |
|  | CBC204 | 14°59'28.9"N 103°36'25.5"E | Elephant Kingdom Surin, Surin | LC789871 |
|  | CBC205 | 14°59'28.9"N 103°36'25.5"E | Elephant Kingdom Surin, Surin | LC789872 |
|  | CBC206 | 14°59'28.9"N 103°36'25.5"E | Elephant Kingdom Surin, Surin | LC789873 |
|  | CBC207 | 14°59'28.9"N 103°36'25.5"E | Elephant Kingdom Surin, Surin | LC789874 |
|  | CBC208 | 14°59'28.9"N 103°36'25.5"E | Elephant Kingdom Surin, Surin | LC789875 |
|  | CBC209 | 14°59'28.9"N 103°36'25.5"E | Elephant Kingdom Surin, Surin | LC789876 |
|  | CBC210 | 14°59'28.9"N 103°36'25.5"E | Elephant Kingdom Surin, Surin | LC789877 |
|  | CBC211 | 14°59'28.9"N 103°36'25.5"E | Elephant Kingdom Surin, Surin | LC789878 |
|  | CBC212 | 14°59'28.9"N 103°36'25.5"E | Elephant Kingdom Surin, Surin | LC789879 |
|  | CBC213 | 14°59'28.9"N 103°36'25.5"E | Elephant Kingdom Surin, Surin | LC789880 |
|  | CBC214 | 14°59'28.9"N 103°36'25.5"E | Elephant Kingdom Surin, Surin | LC789881 |
|  | CBC215 | 14°59'28.9"N 103°36'25.5"E | Elephant Kingdom Surin, Surin | LC789882 |
|  | CBC216 | 14°59'28.9"N 103°36'25.5"E | Elephant Kingdom Surin, Surin | LC789883 |
|  | CBC217 | 14°59'28.9"N 103°36'25.5"E | Elephant Kingdom Surin, Surin | LC789884 |
|  | CBC218 | 14°59'28.9"N 103°36'25.5"E | Elephant Kingdom Surin, Surin | LC789885 |
|  | CBC219 | 14°59'28.9"N 103°36'25.5"E | Elephant Kingdom Surin, Surin | LC789886 |
|  | CBC220 | 14°59'28.9"N 103°36'25.5"E | Elephant Kingdom Surin, Surin | LC789887 |
|  | CBC221 | 14°59'28.9"N 103°36'25.5"E | Elephant Kingdom Surin, Surin | LC789888 |
|  | CBC222 | 14°59'28.9"N 103°36'25.5"E | Elephant Kingdom Surin, Surin | LC789889 |
|  | CBC223 | 14°59'28.9"N 103°36'25.5"E | Elephant Kingdom Surin, Surin | LC789890 |
|  | CBC224 | 14°59'28.9"N 103°36'25.5"E | Elephant Kingdom Surin, Surin | LC789891 |
|  | CBC225 | 14°59'28.9"N 103°36'25.5"E | Elephant Kingdom Surin, Surin | LC789892 |
|  | CBC226 | 14°59'28.9"N 103°36'25.5"E | Elephant Kingdom Surin, Surin | LC789893 |
|  | CBC227 | 14°59'28.9"N 103°36'25.5"E | Elephant Kingdom Surin, Surin | LC789894 |
|  | CBC228 | 14°59'28.9"N 103°36'25.5"E | Elephant Kingdom Surin, Surin | LC789895 |
|  | CBC229 | 14°59'28.9"N 103°36'25.5"E | Elephant Kingdom Surin, Surin | LC789896 |
|  | CBC230 | 14°59'28.9"N 103°36'25.5"E | Elephant Kingdom Surin, Surin | LC789897 |
|  | CBC231 | 14°59'28.9"N 103°36'25.5"E | Elephant Kingdom Surin, Surin | LC789898 |
|  | CBC232 | 14°59'28.9"N 103°36'25.5"E | Elephant Kingdom Surin, Surin | LC789899 |
|  | CBC233 | 14°59'28.9"N 103°36'25.5"E | Elephant Kingdom Surin, Surin | LC789900 |
|  | CBC234 | 14°59'28.9"N 103°36'25.5"E | Elephant Kingdom Surin, Surin | LC789761 |
|  | CBC235 | 14°59'28.9"N 103°36'25.5"E | Elephant Kingdom Surin, Surin | LC789762 |
|  | CBC236 | 14°59'28.9"N 103°36'25.5"E | Elephant Kingdom Surin, Surin | LC789763 |
|  | MEP237 | 19°11'53.7"N 98°53'12.1"E | Maetaeng Elephant Park, Chiang Mai | LC699962 |
|  | MEP238 | 19°11'53.7"N 98°53'12.1"E | Maetaeng Elephant Park, Chiang Mai | LC699963 |
|  | MEP239 | 19°11'53.7"N 98°53'12.1"E | Maetaeng Elephant Park, Chiang Mai | LC699964 |
|  | MEP240 | 19°11'53.7"N 98°53'12.1"E | Maetaeng Elephant Park, Chiang Mai | LC699965 |
|  | MEP241 | 19°11'53.7"N 98°53'12.1"E | Maetaeng Elephant Park, Chiang Mai | LC699966 |
|  | MEP242 | 19°11'53.7"N 98°53'12.1"E | Maetaeng Elephant Park, Chiang Mai | LC699967 |
|  | MEP243 | 19°11'53.7"N 98°53'12.1"E | Maetaeng Elephant Park, Chiang Mai | LC699968 |
|  | MEP244 | 19°11'53.7"N 98°53'12.1"E | Maetaeng Elephant Park, Chiang Mai | LC699969 |
|  | MEP245 | 19°11'53.7"N 98°53'12.1"E | Maetaeng Elephant Park, Chiang Mai | LC699970 |
|  | MEP246 | 19°11'53.7"N 98°53'12.1"E | Maetaeng Elephant Park, Chiang Mai | LC699971 |
|  | MEP247 | 19°11'53.7"N 98°53'12.1"E | Maetaeng Elephant Park, Chiang Mai | LC699972 |
|  | MEP248 | 19°11'53.7"N 98°53'12.1"E | Maetaeng Elephant Park, Chiang Mai | LC699973 |
|  | MEP249 | 19°11'53.7"N 98°53'12.1"E | Maetaeng Elephant Park, Chiang Mai | LC699974 |
|  | MEP250 | 19°11'53.7"N 98°53'12.1"E | Maetaeng Elephant Park, Chiang Mai | LC699975 |
|  | MEP251 | 19°11'53.7"N 98°53'12.1"E | Maetaeng Elephant Park, Chiang Mai | LC699976 |
|  | MEP252 | 19°11'53.7"N 98°53'12.1"E | Maetaeng Elephant Park, Chiang Mai | LC699977 |
|  | MEP253 | 19°11'53.7"N 98°53'12.1"E | Maetaeng Elephant Park, Chiang Mai | LC699978 |
|  | MEP254 | 19°11'53.7"N 98°53'12.1"E | Maetaeng Elephant Park, Chiang Mai | LC699979 |
|  | MEP255 | 19°11'53.7"N 98°53'12.1"E | Maetaeng Elephant Park, Chiang Mai | LC699980 |
|  | MEP256 | 19°11'53.7"N 98°53'12.1"E | Maetaeng Elephant Park, Chiang Mai | LC699981 |
|  | MEP257 | 19°11'53.7"N 98°53'12.1"E | Maetaeng Elephant Park, Chiang Mai | LC699982 |
|  | MEP258 | 19°11'53.7"N 98°53'12.1"E | Maetaeng Elephant Park, Chiang Mai | LC699983 |
|  | MEP259 | 19°11'53.7"N 98°53'12.1"E | Maetaeng Elephant Park, Chiang Mai | LC699984 |
|  | MEP260 | 19°11'53.7"N 98°53'12.1"E | Maetaeng Elephant Park, Chiang Mai | LC699985 |
|  | MEP261 | 19°11'53.7"N 98°53'12.1"E | Maetaeng Elephant Park, Chiang Mai | LC699986 |
|  | MEP262 | 19°11'53.7"N 98°53'12.1"E | Maetaeng Elephant Park, Chiang Mai | LC699987 |
|  | MEP263 | 19°11'53.7"N 98°53'12.1"E | Maetaeng Elephant Park, Chiang Mai | LC699988 |
|  | MEP264 | 19°11'53.7"N 98°53'12.1"E | Maetaeng Elephant Park, Chiang Mai | LC699989 |
|  | MEP265 | 19°11'53.7"N 98°53'12.1"E | Maetaeng Elephant Park, Chiang Mai | LC699990 |
|  | MEP266 | 19°11'53.7"N 98°53'12.1"E | Maetaeng Elephant Park, Chiang Mai | LC699991 |
|  | MEP267 | 19°11'53.7"N 98°53'12.1"E | Maetaeng Elephant Park, Chiang Mai | LC699992 |
|  | MEP268 | 19°11'53.7"N 98°53'12.1"E | Maetaeng Elephant Park, Chiang Mai | LC699993 |
|  | MEP269 | 19°11'53.7"N 98°53'12.1"E | Maetaeng Elephant Park, Chiang Mai | LC699994 |
|  | MEP270 | 19°11'53.7"N 98°53'12.1"E | Maetaeng Elephant Park, Chiang Mai | LC699995 |
|  | MEP271 | 19°11'53.7"N 98°53'12.1"E | Maetaeng Elephant Park, Chiang Mai | LC699996 |
|  | MEP272 | 19°11'53.7"N 98°53'12.1"E | Maetaeng Elephant Park, Chiang Mai | LC699997 |
|  | MEP273 | 19°11'53.7"N 98°53'12.1"E | Maetaeng Elephant Park, Chiang Mai | LC699998 |
|  | MEP274 | 19°11'53.7"N 98°53'12.1"E | Maetaeng Elephant Park, Chiang Mai | LC699999 |
|  | MEP275 | 19°11'53.7"N 98°53'12.1"E | Maetaeng Elephant Park, Chiang Mai | LC700000 |
|  | MEP276 | 19°11'53.7"N 98°53'12.1"E | Maetaeng Elephant Park, Chiang Mai | LC700001 |
|  | MEP277 | 19°11'53.7"N 98°53'12.1"E | Maetaeng Elephant Park, Chiang Mai | LC700002 |
|  | MEP278 | 19°11'53.7"N 98°53'12.1"E | Maetaeng Elephant Park, Chiang Mai | LC700003 |
|  | MEP279 | 19°11'53.7"N 98°53'12.1"E | Maetaeng Elephant Park, Chiang Mai | LC700004 |
|  | MEP280 | 19°11'53.7"N 98°53'12.1"E | Maetaeng Elephant Park, Chiang Mai | LC700005 |
|  | MEP281 | 19°11'53.7"N 98°53'12.1"E | Maetaeng Elephant Park, Chiang Mai | LC700006 |
|  | MEP282 | 19°11'53.7"N 98°53'12.1"E | Maetaeng Elephant Park, Chiang Mai | LC700007 |
|  | BCEP283 | 19°06'50.5"N 98°53'39.3"E | Baan Chang Elephant Park, Chiang Mai | LC700008 |
|  | BCEP284 | 19°06'50.5"N 98°53'39.3"E | Baan Chang Elephant Park, Chiang Mai | LC700009 |
|  | BCEP285 | 19°06'50.5"N 98°53'39.3"E | Baan Chang Elephant Park, Chiang Mai | LC700010 |
|  | BCEP286 | 19°06'50.5"N 98°53'39.3"E | Baan Chang Elephant Park, Chiang Mai | LC700011 |
|  | BCEP287 | 19°06'50.5"N 98°53'39.3"E | Baan Chang Elephant Park, Chiang Mai | LC700012 |
|  | BCEP288 | 19°06'50.5"N 98°53'39.3"E | Baan Chang Elephant Park, Chiang Mai | LC700013 |
|  | BCEP289 | 19°06'50.5"N 98°53'39.3"E | Baan Chang Elephant Park, Chiang Mai | LC700014 |
|  | BCEP290 | 19°06'50.5"N 98°53'39.3"E | Baan Chang Elephant Park, Chiang Mai | LC700015 |
|  | BCEP291 | 19°06'50.5"N 98°53'39.3"E | Baan Chang Elephant Park, Chiang Mai | LC700016 |
|  | BCEP292 | 19°06'50.5"N 98°53'39.3"E | Baan Chang Elephant Park, Chiang Mai | LC700017 |
|  | BCEP293 | 19°06'50.5"N 98°53'39.3"E | Baan Chang Elephant Park, Chiang Mai | LC700018 |
|  | BCEP294 | 19°06'50.5"N 98°53'39.3"E | Baan Chang Elephant Park, Chiang Mai | LC700019 |
|  | BCEP295 | 19°06'50.5"N 98°53'39.3"E | Baan Chang Elephant Park, Chiang Mai | LC700020 |
|  | BCEP296 | 19°06'50.5"N 98°53'39.3"E | Baan Chang Elephant Park, Chiang Mai | LC700021 |
|  | BCEP297 | 19°06'50.5"N 98°53'39.3"E | Baan Chang Elephant Park, Chiang Mai | LC700022 |
|  | BCEP298 | 19°06'50.5"N 98°53'39.3"E | Baan Chang Elephant Park, Chiang Mai | LC700023 |
|  | BCEP299 | 19°06'50.5"N 98°53'39.3"E | Baan Chang Elephant Park, Chiang Mai | LC700024 |
|  | BCEP300 | 19°06'50.5"N 98°53'39.3"E | Baan Chang Elephant Park, Chiang Mai | LC700025 |
|  | BCEP301 | 19°06'50.5"N 98°53'39.3"E | Baan Chang Elephant Park, Chiang Mai | LC700026 |
|  | BCEP302 | 19°06'50.5"N 98°53'39.3"E | Baan Chang Elephant Park, Chiang Mai | LC700027 |
|  | BCEP303 | 19°06'50.5"N 98°53'39.3"E | Baan Chang Elephant Park, Chiang Mai | LC700028 |
|  | BCEP304 | 19°06'50.5"N 98°53'39.3"E | Baan Chang Elephant Park, Chiang Mai | LC700029 |
|  | BCEP305 | 19°06'50.5"N 98°53'39.3"E | Baan Chang Elephant Park, Chiang Mai | LC700030 |
|  | BCEP306 | 19°06'50.5"N 98°53'39.3"E | Baan Chang Elephant Park, Chiang Mai | LC700031 |
|  | BCEP307 | 19°06'50.5"N 98°53'39.3"E | Baan Chang Elephant Park, Chiang Mai | LC700032 |
|  | BCEP308 | 19°06'50.5"N 98°53'39.3"E | Baan Chang Elephant Park, Chiang Mai | LC700033 |
|  | BCEP309 | 19°06'50.5"N 98°53'39.3"E | Baan Chang Elephant Park, Chiang Mai | LC700034 |
|  | BCEP310 | 19°06'50.5"N 98°53'39.3"E | Baan Chang Elephant Park, Chiang Mai | LC700035 |
|  | BCEP311 | 19°06'50.5"N 98°53'39.3"E | Baan Chang Elephant Park, Chiang Mai | LC700036 |
|  | BCEP312 | 19°06'50.5"N 98°53'39.3"E | Baan Chang Elephant Park, Chiang Mai | LC700037 |
|  | BCEP313 | 19°06'50.5"N 98°53'39.3"E | Baan Chang Elephant Park, Chiang Mai | LC700038 |
|  | BCEP314 | 19°06'50.5"N 98°53'39.3"E | Baan Chang Elephant Park, Chiang Mai | LC700039 |
|  | BCEP315 | 19°06'50.5"N 98°53'39.3"E | Baan Chang Elephant Park, Chiang Mai | LC700040 |
|  | BCEP316 | 19°06'50.5"N 98°53'39.3"E | Baan Chang Elephant Park, Chiang Mai | LC700041 |
|  | BCEP317 | 19°06'50.5"N 98°53'39.3"E | Baan Chang Elephant Park, Chiang Mai | LC700042 |
|  | BCEP318 | 19°06'50.5"N 98°53'39.3"E | Baan Chang Elephant Park, Chiang Mai | LC700043 |
|  | BCEP319 | 19°06'50.5"N 98°53'39.3"E | Baan Chang Elephant Park, Chiang Mai | LC700044 |
|  | BCEP320 | 19°06'50.5"N 98°53'39.3"E | Baan Chang Elephant Park, Chiang Mai | LC700045 |
|  | BCEP321 | 19°06'50.5"N 98°53'39.3"E | Baan Chang Elephant Park, Chiang Mai | LC700046 |
|  | BCEP322 | 19°06'50.5"N 98°53'39.3"E | Baan Chang Elephant Park, Chiang Mai | LC700047 |
|  | Wild323 | 13°14'58.0"N 101°41'9.8"E | Khao Ang Rue Nai, Chachoengsao | LC789922 |
|  | Wild324 | − | − | LC789923 |
|  | Wild325 | 12°59'6.7"N 101°42'19.7"E | Rayong | LC789924 |
|  | Wild326 | 12°59'6.7"N 101°42'19.7"E | Rayong | – |
|  | Wild327 | 13°14'58.0"N 101°41'9.8"E | Khao Ang Rue Nai, Chachoengsao | – |
|  | Wild328 | 13°14'58.0"N 101°41'9.8"E | Khao Ang Rue Nai, Chachoengsao | – |
|  | Wild329 | 14°18'40.3"N 101°31'49.7"E | Khao Yai National Park, Nakhon Ratchasima | – |
