# Supplementary material for: Genetic insights for enhancing conservation strategies in captive and wild Asian elephants through improved non-invasive DNA-based individual identification
Source: PLoS One. 2025 May 12;20(5):e0320480. doi: 10.1371/journal.pone.0320480 (PMC12068619; doi:10.1371/journal.pone.0320480)
Supplement: S2 Table — (DOCX) [file pone.0320480.s009.docx]

**S2 Table.** Microsatellite primers and sequences

| Primer | **Fluorescence** |  | **Primer sequence 5′ to 3′** | | **Size (bp)** |
| --- | --- | --- | --- | --- | --- |
|  |  |  | **Reverse** | **Forward** |  |
| LaT06 | FAM |  | CAAAGTGAATCGCCCGTC | GGAACCCTACCAATGCTCTG | 98–126 |
| LaT08 | FAM |  | CATCCATTACAGGAAGACTGC | AGGGTTTGCTTTTCAGGAC | 105–171 |
| LaT16 | HEX |  | GGTGAGCCCAGAGAATCTTC | TGACCCTTTGCTGTATCCTG | 140–142 |
| LaT13 | HEX |  | AAGGTTTGTCCCCACATTC | GAAAAAGAAGCACACACACG | 134–142 |
| LaT17 | FAM |  | GGTGAGCCCAGAGAATCTTC | TGACCCTTTGCTGTATCCTG | 127–131 |
| LaT24 | HEX |  | TTGTTATGCTTGATGTTATTTTGTTAC | GAACTGAACTTGTTAGTATGTTGGG | 156–190 |
| LaT18 | FAM |  | GGCATTTTATTGGGGGAGAG | GGTCTGTGACATTAGTTCCTTTCC | 182–194 |
| LaT25 | HEX |  | CATCCTTTTTCGTGGCTG | AATGAACAAAGAGGGTGTCAC | 102–209 |
| LaT26 | Hex |  | TTTCCTGCTTGAGAGCCAAA | AACCCAGGCTAAAGCACCAA | 352−392 |
| FH1 | Hex |  | ACAGTCTCCCTTGGGAAGAC | GATCAGACCATGGCATGA | 81 |
| FH19 | Fam |  | CTGCATACTCATCGAAGTCACC | GAAGCTCATGGTCAAGGTCAC | 185 |
| FH48 | Hex |  | CCTCCCTGGAATCTGTACAG | GAGTCTCCATAATCAAGAGC | 178 |
| FH65 | Fam |  | CATGAATAAACCCAGCCTCTG | GGCTGTAGCATTTTACACTCCC | 241 |
| FH67 | Fam |  | GGCGTATAGGATAGTTCCAC | GCTTCTCTAGAAATGTGTATGC | 97 |
| FH71 | Hex |  | CTAAGCACATCAGGGAC | GGGATTGGCTAAAATAG | 69 |
| FH94 | Fam |  | ATTGGTTAATTTGCCAGTCCC | TTCCTCCCACAGAGCAGC | 229 |
| FH102 | Hex |  | GTCATTACTGTTATGTTTATTGC | CTTCATTACTGACCTAAACGAG | 179 |
| FH103 | Fam |  | GATGTTGAGACAGTTCTGTAAG | TGTGCTGCCACTTCCTACAC | 154 |
