# Supplementary material for: Genetic insights for enhancing conservation strategies in captive and wild Asian elephants through improved non-invasive DNA-based individual identification
Source: PLoS One. 2025 May 12;20(5):e0320480. doi: 10.1371/journal.pone.0320480 (PMC12068619; doi:10.1371/journal.pone.0320480)
Supplement: S3 Table — (DOCX) [file pone.0320480.s010.docx]

**S3 Table.** Genetic diversity of 329 Asian elephants (*Elephas maximus*) individuals based on 18 microsatellite loci

| **Population** | **Locus** | **N** | ***N*_a_** | ***AR*** | ***N*_e_** | ***I*** | ***H*_o_** | ***H*_e_** | ***F*** | ***M-*ratio** | ***PIC*** |
| --- | --- | --- | --- | --- | --- | --- | --- | --- | --- | --- | --- |
| NEI^1^ | LaT06 | 92 | 36.000 | 36.000 | 15.516 | 3.138 | 0.293 | 0.936 | 0.686 | 0.265 | 0.9324 |
|  | LaT08 | 93 | 27.000 | 26.914 | 8.186 | 2.534 | 0.312 | 0.878 | 0.645 | 0.105 | 0.8676 |
|  | LaT16 | 93 | 29.000 | 28.946 | 9.999 | 2.751 | 0.484 | 0.900 | 0.462 | 0.274 | 0.8934 |
|  | LaT13 | 93 | 21.000 | 20.978 | 8.830 | 2.495 | 0.505 | 0.887 | 0.430 | 0.175 | 0.8774 |
|  | LaT17 | 93 | 15.000 | 14.989 | 5.631 | 2.048 | 0.172 | 0.822 | 0.791 | 0.156 | 0.801 |
|  | LaT24 | 93 | 25.000 | 24.914 | 7.320 | 2.387 | 0.398 | 0.863 | 0.539 | 0.139 | 0.8505 |
|  | LaT18 | 92 | 33.000 | 33.000 | 9.043 | 2.839 | 0.174 | 0.889 | 0.804 | 0.223 | 0.8837 |
|  | LaT25 | 93 | 23.000 | 22.978 | 7.475 | 2.461 | 0.441 | 0.866 | 0.491 | 0.267 | 0.8559 |
|  | LaT26 | 93 | 37.000 | 36.978 | 19.284 | 3.271 | 0.237 | 0.948 | 0.751 | 0.325 | 0.9459 |
|  | FH1 | 93 | 9.000 | 9.000 | 1.828 | 1.088 | 0.409 | 0.453 | 0.098 | 0.409 | 0.4397 |
|  | FH19 | 93 | 8.000 | 7.989 | 2.014 | 1.117 | 0.086 | 0.504 | 0.829 | 0.286 | 0.4816 |
|  | FH48 | 93 | 8.000 | 7.989 | 3.259 | 1.525 | 0.204 | 0.693 | 0.705 | 0.182 | 0.665 |
|  | FH65 | 93 | 12.000 | 11.989 | 2.792 | 1.446 | 0.151 | 0.642 | 0.765 | 0.133 | 0.6035 |
|  | FH67 | 93 | 10.000 | 9.989 | 3.976 | 1.628 | 0.559 | 0.748 | 0.253 | 0.625 | 0.7144 |
|  | FH71 | 92 | 13.000 | 13.000 | 8.250 | 2.219 | 0.750 | 0.879 | 0.147 | 0.433 | 0.8663 |
|  | FH94 | 93 | 11.000 | 10.989 | 5.455 | 1.908 | 0.516 | 0.817 | 0.368 | 0.324 | 0.7933 |
|  | FH102 | 93 | 11.000 | 10.978 | 5.797 | 1.915 | 0.344 | 0.827 | 0.584 | 0.289 | 0.8054 |
|  | FH103 | 93 | 14.000 | 13.968 | 5.979 | 2.040 | 0.398 | 0.833 | 0.522 | 0.438 | 0.8144 |
|  | Mean | 92.833 | 19.000 | 18.977 | 7.258 | 2.156 | 0.357 | 0.799 | 0.548 | 0.217 | 0.7829 |
|  | SE | 0.090 | 2.361 | 9.728 | 1.056 | 0.151 | 0.040 | 0.033 | 0.053 | 0.154 | 0.1425 |
| ESK^2^ | LaT06 | 134 | 46.000 | 36.664 | 15.211 | 3.264 | 0.328 | 0.934 | 0.649 | 0.242 | 0.9316 |
|  | LaT08 | 140 | 47.000 | 40.215 | 23.431 | 3.454 | 0.264 | 0.957 | 0.724 | 0.318 | 0.9557 |
|  | LaT16 | 142 | 28.000 | 27.401 | 9.511 | 2.606 | 0.577 | 0.895 | 0.355 | 0.233 | 0.8861 |
|  | LaT13 | 141 | 41.000 | 35.447 | 15.741 | 3.135 | 0.496 | 0.936 | 0.470 | 0.293 | 0.9331 |
|  | LaT17 | 139 | 48.000 | 41.974 | 5.866 | 2.763 | 0.201 | 0.830 | 0.757 | 0.267 | 0.8234 |
|  | LaT24 | 137 | 30.000 | 28.538 | 9.928 | 2.848 | 0.460 | 0.899 | 0.489 | 0.150 | 0.8943 |
|  | LaT18 | 140 | 33.000 | 24.694 | 10.956 | 2.895 | 0.221 | 0.909 | 0.756 | 0.232 | 0.9038 |
|  | LaT25 | 128 | 43.000 | 40.204 | 20.648 | 3.346 | 0.391 | 0.952 | 0.589 | 0.269 | 0.9495 |
|  | LaT26 | 130 | 36.000 | 32.000 | 17.028 | 3.130 | 0.115 | 0.941 | 0.877 | 0.254 | 0.9383 |
|  | FH1 | 143 | 8.000 | 7.867 | 3.313 | 1.449 | 0.364 | 0.698 | 0.479 | 0.400 | 0.6505 |
|  | FH19 | 143 | 14.000 | 13.600 | 2.283 | 1.399 | 0.224 | 0.562 | 0.602 | 0.077 | 0.5451 |
|  | FH48 | 140 | 21.000 | 15.557 | 6.344 | 2.280 | 0.236 | 0.842 | 0.720 | 0.138 | 0.8254 |
|  | FH65 | 143 | 26.000 | 14.787 | 3.374 | 1.945 | 0.196 | 0.704 | 0.722 | 0.210 | 0.6861 |
|  | FH67 | 140 | 8.000 | 7.802 | 4.506 | 1.638 | 0.857 | 0.778 | −0.102 | 0.333 | 0.7449 |
|  | FH71 | 136 | 12.000 | 10.882 | 2.298 | 1.331 | 0.493 | 0.565 | 0.128 | 0.375 | 0.5432 |
|  | FH94 | 140 | 20.000 | 16.903 | 8.901 | 2.458 | 0.686 | 0.888 | 0.227 | 0.385 | 0.8777 |
|  | FH102 | 129 | 14.000 | 13.952 | 4.661 | 1.902 | 0.403 | 0.785 | 0.487 | 0.389 | 0.7639 |
|  | FH103 | 142 | 11.000 | 6.977 | 4.667 | 1.795 | 0.373 | 0.786 | 0.525 | 0.393 | 0.7568 |
|  | Mean | 138.167 | 27.000 | 23.081 | 9.370 | 2.424 | 0.383 | 0.826 | 0.525 | 0.246 | 0.8116 |
|  | SE | 1.147 | 3.337 | 12.068 | 1.538 | 0.171 | 0.045 | 0.029 | 0.059 | 0.217 | 0.1302 |
| MEP^3^ | LaT06 | 46 | 15.000 | 15.000 | 8.744 | 2.380 | 0.370 | 0.886 | 0.583 | 0.542 | 0.9416 |
|  | LaT08 | 46 | 36.000 | 36.000 | 17.857 | 3.256 | 0.783 | 0.944 | 0.171 | 0.235 | 0.8217 |
|  | LaT16 | 46 | 17.000 | 17.000 | 6.187 | 2.207 | 0.804 | 0.838 | 0.041 | 0.204 | 0.8722 |
|  | LaT13 | 46 | 13.000 | 13.000 | 8.584 | 2.280 | 0.848 | 0.884 | 0.040 | 1.023 | 0.5929 |
|  | LaT17 | 46 | 7.000 | 7.000 | 2.814 | 1.287 | 0.609 | 0.645 | 0.056 | 0.603 | 0.2076 |
|  | LaT24 | 46 | 4.000 | 4.000 | 1.279 | 0.475 | 0.196 | 0.218 | 0.103 | 1.545 | 0.7909 |
|  | LaT18 | 46 | 13.000 | 13.000 | 5.251 | 2.022 | 0.500 | 0.810 | 0.382 | 0.130 | 0.6046 |
|  | LaT25 | 46 | 10.000 | 10.000 | 2.770 | 1.421 | 0.587 | 0.639 | 0.081 | 0.720 | 0.8799 |
|  | LaT26 | 46 | 17.000 | 17.000 | 9.004 | 2.456 | 0.913 | 0.889 | −0.027 | 0.385 | 0.534 |
|  | FH1 | 46 | 4.000 | 4.000 | 2.410 | 1.072 | 0.739 | 0.585 | −0.263 | 4.667 | 0.4103 |
|  | FH19 | 46 | 5.000 | 5.000 | 2.042 | 0.828 | 0.826 | 0.510 | −0.619 | 0.281 | 0.5836 |
|  | FH48 | 46 | 10.000 | 10.000 | 2.792 | 1.349 | 0.935 | 0.642 | −0.457 | 2.333 | 0.6474 |
|  | FH65 | 46 | 5.000 | 5.000 | 3.238 | 1.358 | 0.913 | 0.691 | −0.321 | 2.455 | 0.6745 |
|  | FH67 | 46 | 7.000 | 7.000 | 3.562 | 1.491 | 0.848 | 0.719 | −0.179 | 1.550 | 0.7603 |
|  | FH71 | 46 | 9.000 | 9.000 | 4.771 | 1.740 | 0.761 | 0.790 | 0.037 | 1.889 | 0.8033 |
|  | FH94 | 46 | 11.000 | 11.000 | 5.734 | 1.947 | 0.674 | 0.826 | 0.184 | 0.611 | 0.8093 |
|  | FH102 | 46 | 12.000 | 12.000 | 5.837 | 2.039 | 0.848 | 0.829 | −0.023 | 0.389 | 0.5639 |
|  | FH103 | 46 | 5.000 | 5.000 | 2.684 | 1.186 | 0.696 | 0.627 | −0.109 | 3.667 | 0.6874 |
|  | Mean | 46.000 | 11.111 | 11.111 | 5.309 | 1.711 | 0.714 | 0.721 | −0.018 | 0.629 | 0.1837 |
|  | SE | 0.000 | 1.777 | 7.325 | 0.928 | 0.159 | 0.047 | 0.041 | 0.067 | 0.318 | 0.8758 |
| BCEP^4^ | LaT06 | 40 | 20.000 | 20.000 | 13.734 | 2.803 | 0.075 | 0.927 | 0.919 | 0.137 | 0.9227 |
|  | LaT08 | 40 | 19.000 | 19.000 | 10.667 | 2.653 | 0.000 | 0.906 | 1.000 | 0.207 | 0.8997 |
|  | LaT16 | 40 | 13.000 | 13.000 | 4.301 | 1.860 | 0.150 | 0.768 | 0.805 | 0.107 | 0.739 |
|  | LaT13 | 40 | 16.000 | 16.000 | 7.862 | 2.408 | 0.025 | 0.873 | 0.971 | 0.308 | 0.8629 |
|  | LaT17 | 40 | 7.000 | 7.000 | 3.445 | 1.427 | 0.400 | 0.710 | 0.436 | 0.159 | 0.6601 |
|  | LaT24 | 40 | 14.000 | 14.000 | 6.015 | 2.159 | 0.000 | 0.834 | 1.000 | 0.200 | 0.8169 |
|  | LaT18 | 40 | 12.000 | 12.000 | 6.004 | 2.067 | 0.025 | 0.833 | 0.970 | 0.240 | 0.8144 |
|  | LaT25 | 40 | 10.000 | 10.000 | 2.649 | 1.435 | 0.000 | 0.623 | 1.000 | 0.137 | 0.5909 |
|  | LaT26 | 40 | 25.000 | 25.000 | 19.048 | 3.084 | 0.100 | 0.948 | 0.894 | 0.266 | 0.945 |
|  | FH1 | 40 | 8.000 | 8.000 | 3.738 | 1.634 | 0.000 | 0.733 | 1.000 | 0.174 | 0.7037 |
|  | FH19 | 40 | 7.000 | 7.000 | 4.755 | 1.691 | 0.050 | 0.790 | 0.937 | 0.389 | 0.7585 |
|  | FH48 | 40 | 12.000 | 12.000 | 6.095 | 2.091 | 0.075 | 0.836 | 0.910 | 0.316 | 0.8182 |
|  | FH65 | 40 | 4.000 | 4.000 | 1.444 | 0.639 | 0.250 | 0.308 | 0.187 | 0.500 | 0.2913 |
|  | FH67 | 40 | 4.000 | 4.000 | 2.104 | 0.957 | 0.475 | 0.525 | 0.095 | 0.667 | 0.4679 |
|  | FH71 | 40 | 10.000 | 10.000 | 3.965 | 1.640 | 0.225 | 0.748 | 0.699 | 0.357 | 0.7097 |
|  | FH94 | 40 | 8.000 | 8.000 | 3.796 | 1.524 | 0.575 | 0.737 | 0.219 | 0.074 | 0.6946 |
|  | FH102 | 40 | 6.000 | 6.000 | 4.414 | 1.607 | 0.725 | 0.773 | 0.063 | 0.400 | 0.7395 |
|  | FH103 | 40 | 4.000 | 4.000 | 2.336 | 0.969 | 0.525 | 0.572 | 0.082 | 0.667 | 0.4894 |
|  | Mean | 40.000 | 11.056 | 11.056 | 5.910 | 1.814 | 0.204 | 0.747 | 0.677 | 0.196 | 0.718 |
|  | SE | 0.000 | 1.406 | 5.797 | 1.059 | 0.154 | 0.055 | 0.037 | 0.089 | 0.142 | 0.1661 |
| **Population** | **Locus** | **N** | ***N*_a_** | ***AR*** | ***N*_e_** | ***I*** | ***H*_o_** | ***H*_e_** | ***F*** | ***M-*ratio** | ***PIC*** |
| Wild^5^ | LaT06 | 7 | 9.000 | 6.084 | 6.533 | 2.045 | 0.571 | 0.847 | 0.325 | 0.065 | 0.876 |
|  | LaT08 | 7 | 1.000 | 1.000 | 1.000 | 0.000 | 0.000 | 0.000 | #N/A | 0.064 | 0.877 |
|  | LaT16 | 7 | 7.000 | 5.538 | 6.125 | 1.871 | 0.857 | 0.837 | −0.024 | 0.028 | 0.878 |
|  | LaT13 | 7 | 4.000 | 2.714 | 1.581 | 0.755 | 0.429 | 0.367 | −0.167 | 0.064 | 0.880 |
|  | LaT17 | 7 | 7.000 | 5.538 | 6.125 | 1.871 | 0.571 | 0.837 | 0.317 | 0.053 | 0.884 |
|  | LaT24 | 7 | 4.000 | 3.501 | 3.161 | 1.240 | 1.000 | 0.684 | −0.463 | 0.089 | 0.886 |
|  | LaT18 | 5 | 6.000 | 5.356 | 5.000 | 1.696 | 0.400 | 0.800 | 0.500 | 0.064 | 0.893 |
|  | LaT25 | 7 | 7.000 | 5.538 | 6.125 | 1.871 | 0.571 | 0.837 | 0.317 | 0.073 | 0.894 |
|  | LaT26 | 6 | 7.000 | 5.483 | 5.143 | 1.792 | 0.333 | 0.806 | 0.586 | 0.055 | 0.900 |
|  | FH1 | 7 | 5.000 | 3.972 | 3.500 | 1.390 | 1.000 | 0.714 | −0.400 | 0.044 | 0.904 |
|  | FH19 | 7 | 1.000 | 1.000 | 1.000 | 0.000 | 0.000 | 0.000 | #N/A | 0.089 | 0.923 |
|  | FH48 | 7 | 3.000 | 2.820 | 2.333 | 0.956 | 0.286 | 0.571 | 0.500 | 0.048 | 0.932 |
|  | FH65 | 4 | 3.000 | 3.000 | 2.667 | 1.040 | 0.000 | 0.625 | 1.000 | 0.049 | 0.933 |
|  | FH67 | 7 | 3.000 | 2.930 | 2.649 | 1.035 | 1.000 | 0.622 | −0.607 | 0.031 | 0.938 |
|  | FH71 | 7 | 4.000 | 3.392 | 2.800 | 1.171 | 0.714 | 0.643 | −0.111 | 0.058 | 0.942 |
|  | FH94 | 7 | 2.000 | 1.985 | 1.690 | 0.598 | 0.000 | 0.408 | 1.000 | 0.058 | 0.945 |
|  | FH102 | 7 | 5.000 | 4.294 | 4.083 | 1.494 | 0.571 | 0.755 | 0.243 | 0.005 | 0.946 |
|  | FH103 | 7 | 1.000 | 1.000 | 1.000 | 0.000 | 0.000 | 0.000 | #N/A | 0.065 | 0.950 |
|  | Mean | 7 | 4.389 | 3.619 | 3.473 | 1.157 | 0.461 | 0.575 | #N/A | 0.056 | 0.910 |
|  | SE | 0.000 | 0.567 | 0.404 | 0.458 | 0.159 | 0.086 | 0.070 | #N/A | 0.005 | 0.007 |

Sample size (N); number of alleles (*N*_a_); allelic richness (*AR*); number of effective alleles (*N*_e_); Shannon’s information index (*I*); observed heterozygosity (*H*_o_); expected heterozygosity (*H*_e_); *M-*ratio test (*M-*ratio); polymorphic information content (*PIC*); fixation index (*F*). ^1^NEI = National Elephant Institute of Thailand, Lumphang. ^2^EKS = Elephant Kingdom Surin. ^3^MEP = Maetaeng Elephant Park. ^4^BCEP = Baag Chang Elephant Park. ^5^Wild Elephants = Rayong, Khao Yai and Khao Ang Rue Nai.
