# Supplementary material for: Genetic insights for enhancing conservation strategies in captive and wild Asian elephants through improved non-invasive DNA-based individual identification
Source: PLoS One. 2025 May 12;20(5):e0320480. doi: 10.1371/journal.pone.0320480 (PMC12068619; doi:10.1371/journal.pone.0320480)
Supplement: S4 Table — (DOCX) [file pone.0320480.s011.docx]

**S4 Table.** Distributions of *r* values and *F*_IS_ values for the Asian elephants (*Elephas maximus*)

| **Population 1** | **Population 2** | **Relatedness (*r*)** | | **Inbreeding coefficient (*F*_IS_)** | |
| --- | --- | --- | --- | --- | --- |
|  |  | **Density** | ***p−*value** | **Density** | ***p−*value** |
| NEI | EKS | 0.249558 | 0 | 0.16708 | 0.086023 |
| NEI | MEP | 0.049338 | 0.034594 | 0.685601 | 2.22E−14 |
| NEI | BCEP | 0.293694 | 0 | 0.781452 | 1.43E−17 |
| NEI | Wild | 0.804615 | 3.55E−12 | 0.663594 | 0.002533 |
| NEI | AllPop | 0.147209 | 0 | 0.110403 | 0.33983 |
| EKS | MEP | 0.219415 | 0 | 0.79021 | 1.00E−22 |
| EKS | BCEP | 0.455162 | 0 | 0.761189 | 1.17E−18 |
| EKS | Wild | 0.857143 | 8.46E−14 | 0.605395 | 0.007236 |
| EKS | AllPop | 0.102348 | 0 | 0.127022 | 0.080189 |
| MEP | BCEP | 0.268395 | 0 | 1 | 3.71E−25 |

^1^NEI = National Elephant Institute of Thailand, Lumphang. ^2^EKS = Elephant Kingdom Surin. ^3^MEP = Maetaeng Elephant Park. ^4^BCEP = Baag Chang Elephant Park. ^5^Wild Elephants = Rayong, Khao Yai and Khao Ang Rue Nai.
