# Supplementary material for: Genetic insights for enhancing conservation strategies in captive and wild Asian elephants through improved non-invasive DNA-based individual identification
Source: PLoS One. 2025 May 12;20(5):e0320480. doi: 10.1371/journal.pone.0320480 (PMC12068619; doi:10.1371/journal.pone.0320480)
Supplement: S5 Table — The numbers indicate p-values, with 110 permutations. (DOCX) [file pone.0320480.s012.docx]

**S5 Table.** Pairwise genetic differentiation (*F_ST_*), pairwise *F*_ST_^ENA^ values with ENA correction for null alleles and *R*_ST_ values of Asian elephants (*Elephas maximus*) based on 18 microsatellite loci. The number indicates *p*-values, with 110 permutations

| **Combination** | ***F*_ST_** | ***F*_ST_^ENA^** | ***R*_ST_** |
| --- | --- | --- | --- |
| NEI^1^ x ESK^2^ | 0.063 | 0.055 | 0.264 |
| NEI x MEP^3^ | 0.132 | 0.132 | 0.473 |
| NEI x BCEP^4^ | 0.127 | 0.113 | 0.432 |
| NEI x Wild^5^ | 0.221 | 0.234 | 0.379 |
| ESK x MEP | 0.154 | 0.162 | 0.437 |
| ESK x BCEP | 0.132 | 0.118 | 0.528 |
| ESK x Wild | 0.207 | 0.227 | 0.379 |
| MEP x BCEP | 0.120 | 0.118 | 0.542 |
| MEP x Wild | 0.289 | 0.278 | 0.625 |
| BCEP x Wild | 0.262 | 0.297 | 0.426 |

^1^NEI = National Elephant Institute of Thailand, Lumphang. ^2^EKS = Elephant Kingdom Surin. ^3^MEP = Maetaeng Elephant Park. ^4^BCEP = Baag Chang Elephant Park. ^5^Wild Elephants = Rayong, Khao Yai and Khao Ang Rue Nai.
