# Supplementary material for: Genetic insights for enhancing conservation strategies in captive and wild Asian elephants through improved non-invasive DNA-based individual identification
Source: PLoS One. 2025 May 12;20(5):e0320480. doi: 10.1371/journal.pone.0320480 (PMC12068619; doi:10.1371/journal.pone.0320480)
Supplement: S6 Table — (DOCX) [file pone.0320480.s013.docx]

**S6 Table.** Analysis of molecular variance (AMOVA) results for Asian elephant (*Elephas maximus*) individuals based on 18 microsatellite loci

| **Source of variation** | **df ^1^** | **Sum of squares** | **Variance components** | **Percentage of variation** |
| --- | --- | --- | --- | --- |
| Among populations | 4 | 430.906 | 0.842 | 10 |
| Among individual | 324 | 3647.568 | 3.847 | 47 |
| Within individual | 329 | 1172.500 | 3.564 | 43 |
| Total | 657 | 5250.974 | 8.253 | 100 |

df^1^ =Degree of freedom
