# Supplementary material for: Genetic insights for enhancing conservation strategies in captive and wild Asian elephants through improved non-invasive DNA-based individual identification
Source: PLoS One. 2025 May 12;20(5):e0320480. doi: 10.1371/journal.pone.0320480 (PMC12068619; doi:10.1371/journal.pone.0320480)
Supplement: S7 Table — (DOCX) [file pone.0320480.s014.docx]

**S7 Table.** All source/recipient population comparisons contain the mean migration rates, and 95% confidence intervals determined by BAYESASS using the microsatellite data for Asian elephants (*Elephas maximus*)

| **Migration route** | **Posterior mean of migration rates** | **Standard deviation** |
| --- | --- | --- |
| NEI ^1^ −> NEI | 0.915 | 0.015 |
| ESK ^2^ −> NEI | 0.075 | 0.014 |
| MEP ^3^ −> NEI | 0.003 | 0.003 |
| BCEP ^4^ −> NEI | 0.003 | 0.003 |
| Wild ^5^ −> NEI | 0.003 | 0.003 |
| NEI −> ESK | 0.002 | 0.002 |
| ESK −> ESK | 0.991 | 0.004 |
| MEP −> ESK | 0.002 | 0.002 |
| BCEP −> ESK | 0.002 | 0.002 |
| Wild −> ESK | 0.002 | 0.002 |
| NEI −> MEP | 0.007 | 0.006 |
| ESK −> MEP | 0.007 | 0.006 |
| MEP −> MEP | 0.974 | 0.012 |
| BCEP −> MEP | 0.007 | 0.006 |
| Wild −> MEP | 0.007 | 0.006 |
| NEI −> BCEP | 0.007 | 0.007 |
| ESK −> BCEP | 0.007 | 0.007 |
| MEP −> BCEP | 0.007 | 0.007 |
| BCEP −> BCEP | 0.970 | 0.014 |
| Wild −> BCEP | 0.007 | 0.007 |
| NEI −> Wild | 0.028 | 0.025 |
| ESK −> Wild | 0.028 | 0.026 |
| MEP −> Wild | 0.028 | 0.025 |
| BCEP −> Wild | 0.028 | 0.026 |
| Wild −> Wild | 0.889 | 0.044 |

^1^NEI = National Elephant Institute of Thailand, Lumphang. ^2^EKS = Elephant Kingdom Surin. ^3^MEP = Maetaeng Elephant Park. ^4^BCEP = Baag Chang Elephant Park. ^5^Wild Elephants = Rayong, Khao Yai and Khao Ang Rue Nai.
