# Supplementary material for: Genetic insights for enhancing conservation strategies in captive and wild Asian elephants through improved non-invasive DNA-based individual identification
Source: PLoS One. 2025 May 12;20(5):e0320480. doi: 10.1371/journal.pone.0320480 (PMC12068619; doi:10.1371/journal.pone.0320480)
Supplement: S8 Table — (DOCX) [file pone.0320480.s015.docx]

**S8 Table.** The effective number of immigrants (*N*_m_) of 329 Asian elephants (*Elephas maximus*) from population i into population j per generation for the 18 microsatellite loci

| **i\j** | **NEI** | **ESK** | **MEP** | **BCEP** | **Wild** |
| --- | --- | --- | --- | --- | --- |
| NEI^1^ |  | 1.076 | 0.116 | 0.400 | 0.002 |
| ESK^2^ | 3.284 |  | 0.076 | 0.084 | 0.047 |
| MEP^3^ | 0.815 | 0.468 |  | 0.093 | 0.014 |
| BCEP^4^ | 0.525 | 0.468 | 0.101 |  | 0.005 |
| Wild^5^ | 0.412 | 0.370 | 0.029 | 0.021 |  |

^1^NEI = National Elephant Institute of Thailand, Lumphang. ^2^EKS = Elephant Kingdom Surin. ^3^MEP = Maetaeng Elephant Park. ^4^BCEP = Baag Chang Elephant Park. ^5^Wild Elephants = Rayong, Khao Yai and Khao Ang Rue Nai.
