# Supplementary material for: Genetic insights for enhancing conservation strategies in captive and wild Asian elephants through improved non-invasive DNA-based individual identification
Source: PLoS One. 2025 May 12;20(5):e0320480. doi: 10.1371/journal.pone.0320480 (PMC12068619; doi:10.1371/journal.pone.0320480)
Supplement: S9 Table — (DOCX) [file pone.0320480.s016.docx]

**S9 Table.**  Summary of microsatellite markers selected by the *PIC*+ACO algorithm according to various margin errors.

| **Number of Loci** | **% Error** | **Loci** |
| --- | --- | --- |
| 2 | 7.81% | LaT06, LaT08 |
| 3 | 6.57% | LaT06, LaT08, LaT13 |
| 4 | 5.89% | LaT06, LaT24, LaT25, LaT26 |
| 5 | 5.12% | LaT06, LaT08, LaT13, LaT24, LaT18 |
| **6** | **4.81%** | **LaT06, LaT08, LaT13, LaT17, LaT24, LaT26** |
| 7 | 4.39% | LaT06, LaT08, LaT13, LaT24, LaT18, LaT26, FH67 |
| 8 | 4.06% | LaT06, LaT08, LaT13, LaT17, LaT24, LaT18, LaT25, FH19 |
| 9 | 3.73% | LaT06, LaT08, LaT24, LaT18, LaT25, LaT26, FH48, FH65, FH67 |
| 10 | 3.42% | LaT06, LaT08, LaT16, LaT13, LaT17, LaT18, LaT25, FH1, FH65, FH103 |
| 11 | 3.08% | LaT06, LaT13, LaT17, LaT24, LaT18, LaT25, LaT26, FH19, FH48, FH65, FH94 |
| 12 | 2.71% | LaT06, LaT08, LaT16, LaT17, LaT24, LaT18, LaT26, FH1, FH19, FH65, FH102, FH103 |
| 13 | 2.29% | LaT06, LaT08, LaT16, LaT17, LaT24, LaT26, FH1, FH19, FH48, FH65, FH71, FH102, FH103 |
| 14 | 1.89% | LaT06, LaT08, LaT16, LaT17, LaT18, LaT25, FH1, FH19, FH48, FH65, FH71, FH94, FH102, FH103 |
| 15 | 1.68% | LaT06, LaT16, LaT13, LaT17, LaT24, LaT18, LaT26, FH1, FH19, FH48, FH65, FH67, FH94, FH102, FH103 |
| 16 | 1.28% | LaT06, LaT08, LaT17, LaT24, LaT18, LaT25, LaT26, FH1, FH19, FH48, FH65, FH67, FH71, FH94, FH102, FH103 |
| 17 | 0.79% | LaT06, LaT16, LaT13, LaT17, LaT24, LaT18, LaT25, LaT26, FH1, FH19, FH48, FH65, FH67, FH71, FH94, FH102, FH103 |
