# Supplementary material for: Genetic insights for enhancing conservation strategies in captive and wild Asian elephants through improved non-invasive DNA-based individual identification
Source: PLoS One. 2025 May 12;20(5):e0320480. doi: 10.1371/journal.pone.0320480 (PMC12068619; doi:10.1371/journal.pone.0320480)
Supplement: S10 Table — (DOCX) [file pone.0320480.s017.docx]

**S10 Table.** Matching probability (MP), Exclusion probability (PE), Probability of Identity (P_(ID)_) and Sibling Probability of Identity (P_(ID)sibs_) values per locus for 9 faecal samples of Thai Asian elephant individuals from Kui Buri National Park based on 18 microsatellite loci

| **Locus** | **MP** | **PE** | **P_(ID)_** | **P_(ID)sibs_** |
| --- | --- | --- | --- | --- |
| LaT06 | 9.4×10^−1^ | 0.1250000 | 3.8×10^−1^ | 5.9×10^−1^ |
| LaT08 | 1.8×10^−1^ | 0.3242455 | 1.2×10^−1^ | 4.2×10^−1^ |
| LaT16 | 3.8×10^−1^ | 0.4239969 | 6.8×10^−2^ | 3.8×10^−1^ |
| LaT13 | 1.7×10^−1^ | 0.2993827 | 1.3×10^−1^ | 4.3×10^−1^ |
| LaT17 | 9.4×10^−1^ | 0.1250000 | 3.8×10^−1^ | 5.9×10^−1^ |
| LaT24 | 8.2×10^−1^ | 0.1953125 | 2.1×10^−1^ | 4.9×10^−1^ |
| LaT18 | 5.8×10^−1^ | 0.3872070 | 8.3×10^−2^ | 3.8×10^−1^ |
| LaT25 | 8.2×10^−1^ | 0.1953125 | 2.1×10^−1^ | 4.9×10^−1^ |
| LaT26 | 8.2×10^−1^ | 0.1953125 | 2.1×10^−1^ | 4.9×10^−1^ |
| FH01 | 1.0×10^0^ | 0.0000000 | 1.0×10^0^ | 1.0×10^0^ |
| FH19 | 1.1×10^−1^ | 0.3199588 | 1.1×10^−1^ | 4.3×10^−1^ |
| FH48 | 4.7×10^−1^ | 0.0793762 | 4.1×10^−1^ | 6.5×10^−1^ |
| FH65 | 1.4×10^−1^ | 0.04704895 | 5.6×10^−2^ | 3.5×10^−1^ |
| FH67 | 6.0×10^−2^ | 0.5012003 | 4.8×10^−2^ | 3.5×10^−1^ |
| FH71 | 5.5×10^−1^ | 0.0456104 | 5.1×10^−1^ | 7.3×10^−1^ |
| FH94 | 8.3×10^−1^ | 0.1250000 | 3.8×10^−1^ | 5.9×10^−1^ |
| FH102 | 1.1×10^−1^ | 0.2748628 | 1.5×10^−1^ | 4.5×10^−1^ |
| FH103 | 2.2×10^−1^ | 0.2569921 | 1.6×10^−1^ | 4.5×10^−1^ |
